# The Isolated Brain Microvessel: A Versatile Experimental Model of the Blood-Brain Barrier

**DOI:** 10.3389/fphys.2020.00398

**Published:** 2020-05-07

**Authors:** William M. Pardridge

**Affiliations:** Department of Medicine, University of California, Los Angeles, Los Angeles, CA, United States

**Keywords:** blood-brain barrier, brain capillary, brain endothelium, genomics, proteomics, *in vitro* BBB, transporters, receptors

## Abstract

A versatile experimental model for the investigation of the blood-brain barrier (BBB), including the neuro-vascular unit, is the isolated brain microvessel preparation. Brain microvessels are primarily comprised of endothelial cells, but also include pericytes, pre-capillary arteriolar smooth muscle cells, astrocyte foot processes, and occasional nerve endings. These microvessels can be isolated from brain with a 3 h procedure, and the microvessels are free of brain parenchyma. Brain microvessels have been isolated from fresh animal brain, fresh human brain obtained at neurosurgery, as well as fresh or frozen autopsy human brain. Brain microvessels are the starting point for isolation of brain microvessel RNA, which then enables the production of BBB cDNA libraries and a genomics analysis of the brain microvasculature. Brain microvessels, combined with quantitative targeted absolute proteomics, allow for the quantitation of specific transporters or receptors expressed at the brain microvasculature. Brain microvessels, combined with specific antibodies and immune labeling of isolated capillaries, allow for the cellular location of proteins expressed within the neuro-vascular unit. Isolated brain microvessels can be used as an “*in vitro*” preparation of the BBB for the study of the kinetic parameters of BBB carrier-mediated transport (CMT) systems, or for the determination of dissociation constants of peptide binding to BBB receptor-mediated transport (RMT) systems expressed at either the animal or the human BBB. This review will discuss how the isolated brain microvessel model system has advanced our understanding of the organization and functional properties of the BBB, and highlight recent renewed interest in this 50 year old model of the BBB.

## Introduction

**FIGURE 1:**
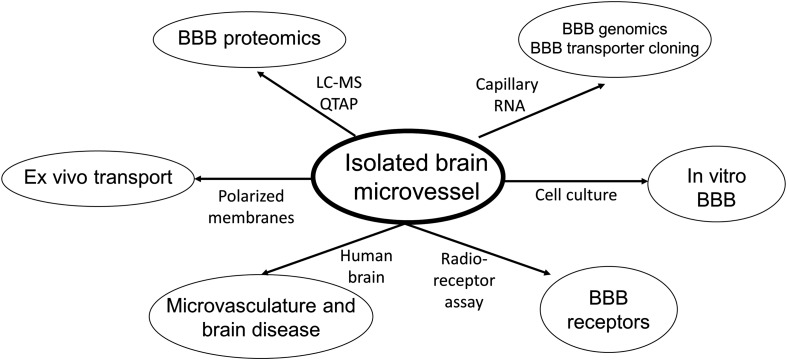
Pathways of investigation following the isolation of microvessels from animal or human brain. LC-MS, liquid chromatography-mass spectrometry; QTAP, quantitative targeted absolute proteomics.

The blood-brain barrier (BBB) restricts the free diffusion of nutrients, hormones, and pharmaceuticals between blood and brain in either the blood-to-brain direction, or the brain-to-blood direction. The cell in brain that limits BBB permeability is the brain capillary endothelium, which is comprised of 2 membrane barriers in series: the luminal and abluminal endothelial plasma membranes. The capillary endothelium is also part of a multi-cellular neurovascular unit (NVU). There are multiple experimental models for the investigation of BBB transport and regulation of the NVU. Of these models, the most versatile is the isolated brain microvessel. Subsequent to the isolation of brain microvessels, these structures can be used in a multitude of applications (Figure 1), including genomics, proteomics, cultured endothelium and *in vitro* BBB models, and biochemical investigations of BBB carrier-mediated transporters (CMT) and receptor-mediated transporters (RMT); the isolation of microvessels from human brain can provide the basis for understanding the role of the brain microvasculature in the etiology of neurological disease. This review will discuss progress in the multiple applications of the isolated brain microvessel in the diverse fields shown in Figure 1.

## Neurovascular Unit

**FIGURE 2:**
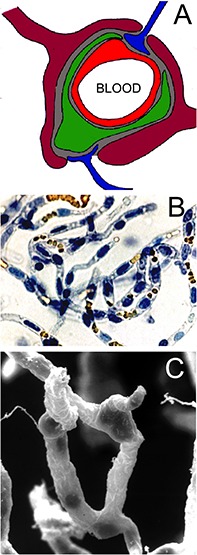
**(A)** Neurovascular unit is comprised of capillary endothelium (red), mural cells such as pericytes (green) or smooth muscle cells, which share a common basement membrane (gray) with the endothelium, astrocyte foot processes (purple), which invest ∼99% of the basement membrane surface, and occasional nerve endings (blue), which directly innervate the microvascular surface. Reprinted by permission from Pardridge (2007). **(B)** Microvessels isolated from fresh bovine brain and stained with trypan blue. The endothelial nuclei are trypan blue positive, and the erythrocytes trapped in the lumen of the capillary are yellow. Reprinted by permission from Boado et al. (1999); copyright 1999 National Academy of Sciences. **(C)** Scanning electron micrograph of bovine brain microvessels show nerve endings attached to the microvessel. Reprinted by permission from Pardridge (2001).

The brain capillary endothelium is part of the NVU as depicted in Figure 2A. The endothelium (red in Figure 2A) shares a microvascular basement membrane (gray in Figure 2A) with a mural cell, the pericyte (green in Figure 2A), or the smooth muscle cell in pre-capillary arterioles. The pericyte covers about one-third of the abluminal surface of the capillary endothelium (Mathiisen et al., 2010). The astrocyte foot process (purple in Figure 2A) invests the microvascular basement membrane. The brain microvessel is directly innervated by neurons (blue in Figure 2A). Kacem et al. (1998), using glial fibrillary acidic protein (GFAP) confocal microscopy, suggested the encasement of the brain microvessel by the astrocyte foot process was incomplete. However, 3-dimensional electron microscopic reconstruction of the NVU in brain shows the basement membrane on the abluminal side of the brain microvessel is >99% invested by astrocyte foot processes, which are separated by clefts of 20−50 nm in diameter (Mathiisen et al., 2010). Since plasma proteins such as the 70 kDa albumin have a molecular diameter of ∼5 nm, large molecules are able to freely move through the clefts formed by the astrocyte foot processes (Thrane et al., 2014). The foot process and the capillary endothelium/pericyte are separated by a distance of only 20 nm *in vivo* (Paulson and Newman, 1987; Mathiisen et al., 2010), and this small space is filled with the capillary basement membrane. The basement membrane is comprised of two layers, an outer, thicker layer closer to the astrocyte foot process, and an inner, thinner layer closer to the endothelium/pericyte (Simard et al., 2003). The brain microvessel includes both capillaries and pre-capillary arterioles, and the basement membrane invests the endothelial cells and the mural cells (pericytes or smooth muscle cells). The nearly complete encasement of the brain microvessel by the astrocyte foot processes is interrupted when there is direct neuronal innervation of the surface of the endothelium/pericyte or smooth muscle cell (Paspalas and Papadopoulos, 1996).

Nearly all elements of the NVU are incorporated in the isolated brain microvessel. The microvessels include both capillaries and pre-capillary arterioles. Microvessel capillaries are shown in Figure 2B; these vessels were isolated from bovine brain and both the endothelium and the pericyte are encased in the basement membrane invested capillary. The nuclei of the endothelium are oval shaped and the nuclei of the pericyte are more circular; orange-colored erythrocytes are seen trapped in the vascular lumen (Figure 2B). A scanning electron micrograph of the isolated brain capillary shows nerve endings adhered to the basement membrane surface of the microvessel (Figure 2C). Glutamergic, serotoninergic, purinergic, and adrenergic nerve endings have been localized to the brain microvessel *in vivo* (Cohen et al., 1995; Paspalas and Papadopoulos, 1996; Parri and Crunelli, 2003; Simard et al., 2003).

### Pericytes

**FIGURE 3:**
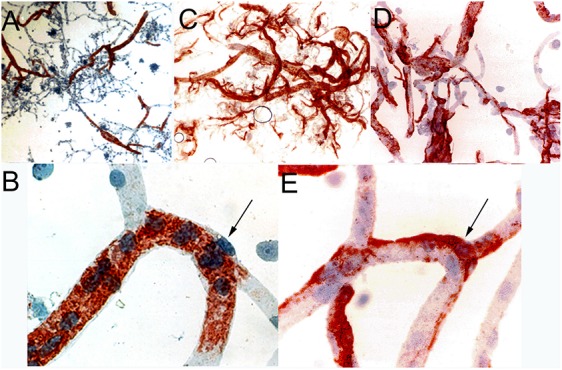
(**A**, low magnification) and (**B**, high magnification) are cytocentrifuged bovine brain microvessels immune-stained with a mouse monoclonal antibody directed against the amino terminal decapeptide of alpha-actin. Only pre-capillary arteriolar smooth muscle cells are immune-positive and capillary pericytes (arrow, panel **B**) are immune-negative. Panels **(A,B)** reprinted by permission from Boado and Pardridge (1994). Panels **(C–E)** are cyto-centrifuged human brain microvessels immune-stained with a mouse monoclonal antibody against the human class II DR antigen. Panel **(C)** shows microvessels from non-multiple sclerosis (MS) human brain, and panels **(D,E)** are microvessels isolated from white matter of MS brain. In non-MS human brain, DR immunoreactivity is confined to pre-capillary arteriolar smooth muscle cells (panel **C**), whereas in MS brain (panels **D,E**), DR immunoreactivity is observed in capillary pericytes (arrow, panel **E**). Panels **(C–E)** reprinted by permission from Pardridge et al. (1989). All slides lightly counter-stained with hematoxylin. Magnifications in panels **(A,C)** are 50X, in panels **(B,E)** are 500X, and in panel **(D)** is 200X.

The capillary pericyte and endothelial cell share a common basement membrane. The pericyte is formed by a cell body with thin processes that extend along the length of the capillary. Such processes have been visualized with two-photon microscopy (Berthiaume et al., 2018). The pericyte processes can extend >100 microns along the capillary abluminal surface, and exhibit a dynamic mobility as compared to the pericyte cell bodies (Berthiaume et al., 2018). Pericytes are said to act as contractile cells at the brain microvessel (Neuhaus et al., 2017), as alpha-actin mRNA is detected in pericytes grown in cell culture (DeNofrio et al., 1989). However, immunoreactive alpha-actin in pericytes is not visualized in immunocytochemistry of freshly isolated brain microvessels. Brain microvessel pericytes and smooth muscle cells were visualized with immunocytochemistry of cyto-centrifuged microvessels followed by immune staining with an antibody to the smooth muscle-specific alpha actin (Boado and Pardridge, 1994). Alpha actin immunoreactivity is only detected in smooth muscle cells of pre-capillary arterioles, and no immunoreactivity is observed in pericytes at the capillary level (Figures 3A,B). The concept persists that the contractile function of the brain pericyte plays a role in the control of cerebral blood flow (Cheng et al., 2018). However, the contractile function, at least under normal conditions, of the pericyte is likely minimal in that the capillary pericyte is immune-negative for alpha-actin (arrow, Figure 3B). Cerebral blood flow is regulated primarily by the smooth muscle cell at the brain microvasculature (Hill et al., 2015). Pericytes may play a role in antigen presentation at the brain microvasculature in neurological disease such as multiple sclerosis (MS), which is mediated by the human leukocyte antigen (HLA)-DR isotype. Immunoreactive HLA-DR antigen at the human brain microvasculature was examined with cyto-centrifuged human brain microvessels and a monoclonal antibody against the HLA-DR antigen (Pardridge et al., 1989). In microvessels from non-MS brain, immunoreactive HLA-DR antigen was detected only in pre-capillary arteriolar smooth muscle cells (Figure 3C). However, HLA-DR antigen immunoreactivity was detected in capillaries isolated from white matter of MS brain (Figure 3D). Higher magnification shows the classic shape of a capillary pericyte, which is immunopositive for the HLA-DR antigen (arrow, Figure 3E). This immunocytochemical study of isolated human brain capillaries shows the extension of capillary pericytes along the abluminal surface of the brain capillary (Figure 3E). Microvessels have been isolated from the brain of multiple subjects with either MS or non-MS neurologic conditions, and HLA-DR immunoreactivity in pericytes was observed in 50% of capillaries isolated from MS plaque, whereas capillaries from non-MS brain exhibited no HLA-DR immunoreactivity (Washington et al., 1994).

### Astrocyte Foot Processes

The isolated brain microvessel contains remnants of astrocyte foot processes, which remain adhered to the basement membrane surface of the vessel. Astrocyte foot processes in isolated brain capillaries were detected with immunocytochemistry and an antibody to GFAP (White et al., 1981). The GFAP mRNA was also detected in mRNA derived from isolated brain microvessels (Li et al., 2002). This surprising finding suggested the GFAP transcript was transported from the cell body to the astrocyte foot process. Confocal microscopy and fluorescent *in situ* hybridization of mouse brain demonstrates the presence in microvascular astrocyte foot processes of mRNA for GFAP, aquaporin-4 (Aqp4), and other astrocyte-specific transcripts (Boulay et al., 2017). Live imaging of brain shows the microtubule-based transport of astrocyte mRNAs and mRNA binding proteins over a distance >100 microns from the cell body (Pilaz et al., 2016). The role of the astrocyte endfeet in the regulation of the NVU has been examined with two photon microscopy of brain following the intra-cerebral injection of a convulsant, 4-aminopyridine (Zhang et al., 2019). Toxin injection caused large increases in cytosolic calcium of astrocyte endfeet, which was associated with vasodilation of pre-capillary arterioles and increased blood flow (Zhang et al., 2019).

### Oligodendrocytes

The oligodendrocyte (OLG), which produces myelin, is generally not associated with the brain microvessel or NVU. However, a surprising finding was the presence of the transcript encoding myelin basic protein (MBP) in the transcriptome of capillaries isolated from rat brain (Li et al., 2001a; Enerson and Drewes, 2006). Recent work indicates that OLG precursor cells use the microvessel as a scaffold for migration, in close proximity to microvascular pericytes, which suggests a role for the pericyte in CNS regeneration (De La Fuente et al., 2017).

### Endothelial Glycocalyx

The luminal membrane of the brain capillary endothelium is lined by the glycocalyx, which is formed by transmembrane proteoglycans extending from the luminal plasma membrane, and many soluble proteins are attached to the core glycocalyx of endothelial cells (Reitsma et al., 2007). The soluble components of the endothelial glycocalyx include glycosaminoglycans, such as hyaluronic acid, heparan sulfate, dermatan sulfate, chondroitin sulfate, and keratan sulfate, as well as proteins with enzymatic function, such as superoxide dismutase, or coagulation factors such as anti-thrombin III (Reitsma et al., 2007). The glycocalyx of the brain capillary is more developed than the glycocalyx of continuous capillaries in peripheral organs, such as in heart or lung. This is shown by measurements of the thickness of the endothelial glycocalyx in these organs. The percent area of the luminal endothelial membrane that is covered by the glycocalyx is 40%, 15%, and 3.7%, respectively, in brain, heart, and lung capillaries (Ando et al., 2018). The thickness of the glycocalyx varies from 100 nm, as measured with transmission electron microscopy of fixed brain (Vogel et al., 2000) to ∼400 nm, as measured by two-photon microscopy of unfixed brain (Yoon et al., 2017). A glycocalyx of 400 nm in depth, which is comprised of a complex mesh of soluble and insoluble proteins, would be expected to provide some kind of barrier function, and this was confirmed with two-photon microscopy of brain. Small molecules, with a molecular weight (MW) of 376−643 Daltons (Da) penetrated the glycocalyx faster than large molecules, with a MW of 40−150 kDa (Kutuzov et al., 2018).

### Capillary Basement Membrane

The protein profile of the brain capillary basement membrane, and extracellular matrix (ECM), was examined with a non-targeted proteomics approach in mouse brain (Chun et al., 2011). Protein abundance was estimated as a spectral count with mass spectrometry. At least 30 ECM proteins were identified in the freshly isolated mouse brain microvessel, and the proteins with the top 10 spectral counts included contactin-1 (Cntn1), perlecan, heparan sulfate proteoglycan (Hspg2), contactin associated protein-1 (Cntnap1), the tenascin receptor (Tnr), laminin α1, α2, β1, β2, α5 (Lamc1, Lama2, Lamb1, Lamb2, Lama5), agrin (Agrn), and nidogen-1 (Nid1) (Chun et al., 2011). The functions of this diverse set of basement membrane proteins is poorly understood, but these proteins undoubtedly play crucial functions in the regulation of the NVU and brain function. For example, in the Nid1-knockout mouse, structural changes in the microvessel are observed only in the brain and the lens capsule, and these mice have neurologic deficits, including seizures (Dong et al., 2002).

The NVU is regulated by the coordinated interactions between multiple cells at the brain microvasculature, as well as the microvessel basement membrane. However, the endothelium solely limits BBB permeability and transport between blood and brain. Neither the capillary basement membrane nor the astrocyte foot process constitutes a permeability hindrance between blood and brain. This was demonstrated by the classical studies of Brightman et al. (1970). Horseradish peroxidase (HRP), which has a molecular weight of 43,000 Da, was injected into the mouse brain and the distribution of the HRP was followed by electron microscopic histochemistry. The HRP was observed to move freely past both the basement membrane and the astrocyte foot process, and diffusion was not blocked until the HRP reached the endothelial abluminal membrane. The lack of a role of the astrocyte foot process in control of BBB permeability was also demonstrated in rats administered the gliotoxin, 6-aminonicotinamide. Despite the toxin-induced loss of astrocyte foot processes, the BBB was still impermeable to blood borne horseradish peroxidase (Krum and Rosenstein, 1993).

## History of Isolated Brain Microvessels

The first isolation of microvessels from brain (bovine and human) was reported by Siakotos and Rouser (1969). Brain was mechanically homogenized, and microvessels were purified with gel filtration followed by separation of brain nuclei and brain microvessels by passage through a glass bead column, and elution of the microvessels, which adhered to the glass beads. Phase contrast microscopy demonstrated purified microvessel tree-like structures, which were held together by the basement membrane. The use of nylon mesh as sieves to capture the microvessels was introduced, and the metabolic activity of the microvessels was demonstrated, as these structures combusted nutrients, such as glucose, pyruvate, glutamate, and oleic acid (Joo and Karnushina, 1973; Brendel et al., 1974; Goldstein et al., 1975). Brain microvessels were shown to take up large neutral amino acids via a saturable carrier-mediated process (Hjelle et al., 1978). However, isolated brain microvessels were subsequently shown to be metabolically impaired, as demonstrated by the observation that microvessels produced with a mechanical homogenization procedure did not exclude trypan blue, a widely used index of cell viability; conversely, microvessels isolated with an enzymatic homogenization were said to exclude trypan blue (Williams et al., 1980). However, intracellular ATP was shown to be <10% of normal values in brain microvessels isolated by either a mechanical or an enzymatic homogenization procedure (Lasbennes and Gayet, 1984). Conversely, intracellular ATP was normal in brain capillary endothelial cells grown in tissue culture (Sussman et al., 1988).

The cause of the low intracellular ATP in freshly isolated brain microvessels has never been elucidated, but may be aberrant intra-endothelial signal transduction related to the disruption of the normal cellular architecture of the NVU *in vitro*. Nevertheless, isolated brain microvessels can be used as an *in vitro* model of BBB carrier-mediated transport (CMT) of nutrients such as amino acids (Hjelle et al., 1978). Amino acid transport kinetics can even be quantified in microvessels isolated from autopsy human brain (Choi and Pardridge, 1986), and the kinetics of large neutral amino acid uptake by human brain microvessels *in vitro* correlated with the kinetic parameters of large neutral amino acid transport across the rat BBB *in vivo* (Hargreaves and Pardridge, 1988). Isolated brain microvessels may be used to quantify the binding of peptides to receptor-mediated transport (RMT) systems using radiolabeled peptides and standard radio-receptor assays (RRA). Capillaries isolated from human autopsy brain were used as the receptor source for RRAs to characterize the receptor on the human BBB for insulin, transferrin, the insulin-like growth factors (IGFs), and leptin (Pardridge et al., 1985a, 1987a; Duffy et al., 1988; Golden et al., 1997). Therefore, despite the metabolic impairment of the isolated brain microvessel, these structures can be used to characterize CMT and RMT systems at the BBB, including the human BBB (see below in section “*Ex vivo* Transport and Receptor Binding in Isolated Brain Microvessels”).

The isolation of brain microvessels enables the production of brain microvessel plasma membranes and plasma membrane vesicles. Such membrane vesicles are polarized in that the vesicle may originate from either the luminal or abluminal endothelial membrane. Isolated bovine brain microvessels were briefly digested with collagenase and mechanically homogenized followed by membrane separation on a discontinuous Ficoll gradient (Betz et al., 1980). This method was subsequently used to investigate the polarity of amino acid transport into luminal vs abluminal vesicles derived from the isolated brain microvessel (Sanchez del Pino et al., 1992). More recently, quantitative targeted absolute proteomics (QTAP) has been used to measure the absolute expression, in pmol per mg capillary protein, of transporters expressed on the luminal vs abluminal membranes of the brain microvessel (Kubo et al., 2015). A model transporter exclusively localized to the luminal membrane is P-glycoprotein (MDR1/Abcb1), and a model transporter exclusively localized to the abluminal membrane is sodium dependent small neutral amino acid A-type transporter (Ata2, Slc38a2) (Kubo et al., 2015).

An analysis of the BBB transcriptome is not possible with a whole brain approach. This is because the volume of the capillary endothelium in brain is 0.1% of the brain volume. Therefore, a BBB genomics program starts with the isolation of RNA derived from isolated brain capillaries. The first BBB genomics study employed suppression subtractive hybridization (SSH) and polyA + RNA purified from capillaries isolated from either rat brain (Li et al., 2001a, 2002) or human brain (Shusta et al., 2002a). One limitation of the original studies is that the transcriptome of all cells comprising the NVU were pooled when the RNA is isolated from intact brain microvessels. More recent work, reviewed below, used fluorescent activated cell sorting (FACS) to separate the transcriptome of brain microvascular endothelial cells or pericytes (Daneman et al., 2010; Chasseigneaux et al., 2018; Munji et al., 2019).

Quantitative BBB proteomics was first developed by subjecting isolated brain microvessels to enzymatic digestion with trypsin, and measurement of capillary derived tryptic peptides by liquid chromatography mass spectrometry (LC-MS), a process called Quantitative Targeted Absolute Proteomics or QTAP (Uchida et al., 2011). Quantitation of the LC-MS signal for a given peptide was enabled by the synthesis of sequence-specific peptide standards, which encoded the amino acid sequence specific to a known gene. These peptide standards encompassed sequence bordered on either side by cationic amino acid residues (lysine, arginine), which are the target sites of trypsin proteolytic cleavage.

## Brain Microvascular Proteomics

The development of BBB drug delivery strategies requires information on the relative expression of transporters and receptors that constitute the carrier-mediated transporters (CMT), which are generally members of the Solute Carrier (SLC) gene family, active efflux transporters (AET), which are generally members of the ATP Binding Cassette (ABC) gene family, and receptor-mediated transporters (RMT). Using QTAP methodology, the absolute expression of many transporters and receptors in freshly isolated capillaries was quantified for human brain (Shawahna et al., 2011; Uchida et al., 2011), mouse brain (Uchida et al., 2013), and rat and marmoset brain (Hoshi et al., 2013). The QTAP methodology has recently been extended to the dog choroid plexus (Braun et al., 2017), and the pig meninges, which includes the blood-arachnoid barrier or BAB (Uchida et al., 2020).

The QTAP methodology is considered targeted proteomics, because peptide sequence specific standards are used in the LC-MS methodology (Uchida et al., 2011). Untargeted quantitative proteomics methodology has been applied to microvessels isolated from frozen rat brain, and this label-free form of LC-MS correlates well with the QTAP approach (Al Feteisi et al., 2018). QTAP methodology has been extended to microvessels isolated from brain bank frozen human brain (Billington et al., 2019).

The quantitative expression of selected AET, CMT, and RMT transporters and receptors, determined with the QTAP methodology, has been reported for brain microvessels or choroid plexus from human, mouse, rat, or dog brain, and only a small fraction of the available QTAP data is summarized in Table 1. As suggested by Ohtsuki et al. (2014), such data provide a platform for pharmaco-proteomics of BBB transport. The caveat is that BBB transport is determined solely by the capillary endothelial cell, whereas the brain microvessel is comprised of all cells that make up the NVU, including pre-capillary arteriolar smooth muscle cells, pericytes, astrocyte foot processes, neuronal endings, and even oligodendrocytes. An astrocyte-specific marker is GFAP, and GFAP content is quite high in isolated brain microvessels (Table 1). This finding was anticipated by early work showing that isolated brain microvessels are immunoreactive with antibodies against GFAP (White et al., 1981), owing to adherence of astrocyte foot processes on the basement membrane surface of the microvessel. Microvessels are also comprised of pericytes. Pericyte-specific genomics studies show the NG2 proteoglycan, also called chondroitin sulfate proteoglycan 4, or Cspg4, is selectively expressed by pericytes (Chasseigneaux et al., 2018), and the Cspg4 protein is detected in isolated brain microvessels (Table 1). An unexpected observation of early BBB transcriptome studies was the finding of myelin basic protein (MBP) mRNA in isolated rat brain microvessels (Li et al., 2001a), and the oligodendrocyte-specific protein, 2’,3’-cyclic nucleotide 3’-phosphodiesterase (CNPase) is detected in brain microvessels (Table 1). Brain microvessels are directly innervated by neuronal axons (Figure 1C), and the synaptic vesicle protein p38, also called synaptophysin (SYP), is detected in isolated brain microvessels (Table 1).

**TABLE 1:** Protein expression level (pmol per mg protein) in brain microvessels and choroid plexus.

	**Brain microvessel**					**Choroid plexus**
**Gene**	**Alias**	**Human**	**Mouse**	**Rat**	**Dog**	**Dog**
**ABC transporters**						
ABCB1	P-gp	6.1	17.8	19.1	6.2	0.14
ABCG2	BCRP	8.1	5.5	5.0		<LOQ
**SLC transporters**						
SLC2A1	GLUT1	139	101	91	194	57
SLC7A5	LAT1	0.43	1.2	3.0	<LOQ	<LOQ
SLC3A2	4F2hc	3.5			23.3	5.8
SLC7A1	CAT1	1.1				
SLC16A1	MCT1	2.3	13.7	12.6	<LOQ	1.5
SLC1A3	EAAT1	25				
**Receptors**						
INSR	IR	1.1	1.1	1.0	<LOQ	0.57
TFRC	TfR1	2.3	5.2	7.8	16.2	9.1
**Structural proteins/enzymes**						
CLDN5	Claudin-5	3.6	8.1	7.5		
GGT1	gGTP	2.1	3.2	3.3		
**Non-endothelial cell proteins^a^**						
GFAP	GFAP	503				
CSPG4	NG2	1.1				
CNP	CNPase	13				
SYP	p38	1.5				

*From Shawahna et al. (2011), Uchida et al. (2011, 2013), Hoshi et al. (2013), and Braun et al. (2017); LOQ, limit of quantitation. ^*a*^GFAP, glial fibrillary acidic protein is an astrocyte marker; CSPG4, chondroitin sulfate proteoglycan 4, also known as NG2 proteoglycann is a pericyte marker; CNP, 2’,3’-cyclic nucleotide 3’-phosphodiesterase is an oligodendrocyte marker; SYP, synaptophysin is a nerve ending marker.*

Despite the cellular heterogeneity of the isolated brain microvessel, very useful information is produced with the QTAP measurements of transporter and receptor expression in brain microvessels, and a partial sampling of this information is highlighted in Table 1:

• P-glycoprotein (Pgp) expression is high at the BBB in all species examined, but the expression of Pgp at the choroid plexus is only 2% of the Pgp expression at the brain microvessel (Table 1). Drug distribution into CSF is often used as an index of drug penetration across the BBB. However, drug movement from blood to CSF is a function of drug transfer across the choroid plexus, which forms the blood-CSF barrier *in vivo*. Conversely, drug penetration into brain parenchyma is a function of transport across the brain capillary endothelium, which forms the BBB *in vivo*. The brain capillary endothelium and the choroid plexus epithelium may have very different transporter/receptor expression profiles. The very low expression of Pgp at the choroid plexus (Table 1) explains why the administration of a Pgp inhibitor increases brain uptake of nelfinavir, a Pgp substrate, but causes no change in the distribution of nelfinavir into CSF (Kaddoumi et al., 2007). These findings show the limitations of using drug penetration into CSF as an index of drug transport into brain parenchyma through the BBB (Pardridge, 2016).
• The expression of the insulin receptor (INSR) and the type 1 transferrin receptor (TFRC) at the human brain microvessel is comparable, but the expression of the Tfrc at the rodent microvessel is 5- to 8-fold higher than the expression of the rodent Insr (Table 1). For this reason, a monoclonal antibody (MAb) against the mouse or rat transferrin receptor (TfR) is the preferred molecular Trojan horse for drug delivery to mice or rats, whereas a MAb against the human insulin receptor has been developed as a molecular Trojan horse for drug delivery across the human BBB (Pardridge and Boado, 2012).
• The expression of the Tfrc at the choroid plexus is 16-fold higher than the expression of the Insr (Table 1). Therefore, a TfRMAb is expected to distribute rapidly into CSF from blood, which has been demonstrated for the Rhesus monkey (Pardridge et al., 2018).
• The GLUT1 glucose transporter is by far the most abundant CMT system at the brain microvessel across all species (Table 1). GLUT1 is a brain capillary endothelial cell-specific marker, as GLUT1 mRNA is not detected in brain parenchyma (Pardridge et al., 1990a). There are over 10 members of the GLUT glucose transporter gene family and various members other than GLUT1 have been implicated as playing a role in BBB glucose transport. However, as discussed below in the *Ex Vivo* Transport section, >95% of all glucose transporter binding sites in the brain microvessel can be accounted for by GLUT1.
• GLUT1 expression at the brain microvessel is about 100-fold more abundant than is the expression of the LAT1 large neutral amino acid transporter type 1 (Table 1). The LAT1 transporter is selectively expressed in brain at the BBB (Boado et al., 1999). This ∼100-fold greater expression of GLUT1, as compared to LAT1, parallels the maximal transport rate, or Vmax, of these transporters at the BBB. The Vmax of BBB transport of glucose is ∼65-fold higher than is the Vmax of BBB transport of phenylalanine (Pardridge, 2012).

Blood-brain barrier pharmaco-proteomics investigations are very useful for understanding drug and nutrient transport at either the BBB or blood-CSF barrier. However, such investigations are limited to known transporters and receptors. The discovery of novel transporters or receptors expressed at the BBB requires the development of alternative uses of the isolated brain capillary, including BBB genomics and expression cloning of BBB cloned RNA or transporter gene cDNAs.

## Brain Microvascular Genomics

**FIGURE 4:**
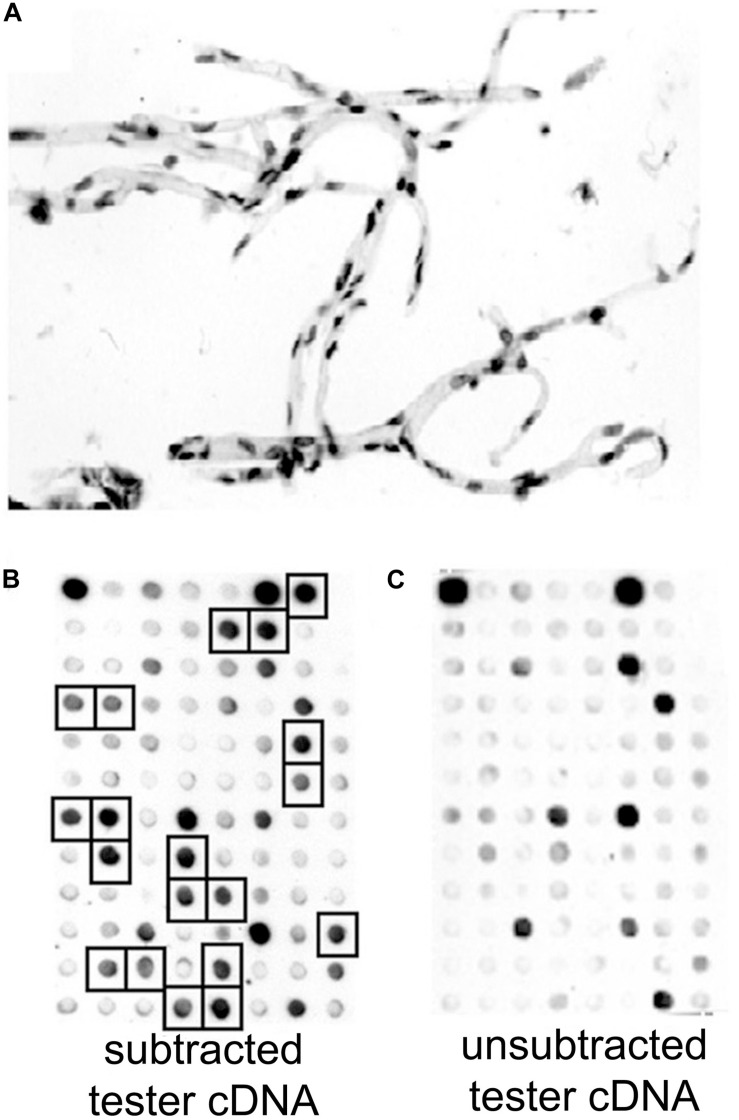
**(A)** Brain microvessels were isolated from fresh human brain removed at neurosurgery for cerebral dysplasia, and stained with toluidine blue. RNA was isolated from the human brain microvessels, and microvascular-enriched genes were isolated by suppression subtractive hybridization. **(B,C)** A 96-well microarray analysis of subtracted tester cDNA library. Filters containing identical arrays of individual subtracted clones were probed with [^32^P]-labeled subtracted tester cDNA **(B)** or unsubtracted tester cDNA **(C)**. Brain microvascular-specific genes present in high levels in the subtracted tester cDNA, and selected for DNA sequencing, are identified with squares in panel **(B)**. Reproduced by permission from Shusta et al. (2002a).

A “BBB genomics” study, or a brain microvascular genomics study, was initially performed using suppression subtractive hybridization (SSH) technology and polyA + RNA purified from freshly isolated rat brain microvessels (Li et al., 2001a, 2002). SSH is a PCR-based open architecture genomics approach (Shackel et al., 2003), and enables amplification of transcripts in a targeted (tester) transcriptome, e.g., the rat brain capillary, relative to a control (driver) transcriptome, e.g., rat liver and kidney. Unlike the use of chip technology, which restricts testing to known genes, the SSH approach enables the cloning of cDNAs encoding novel genes selectively expressed at the brain capillary. The SSH approach to BBB genomics was expanded to the human brain, using fresh human brain tissue removed at neurosurgery for intractable epilepsy caused by cerebral dysplasia (Shusta et al., 2002a). The microvessels derived from fresh human brain are free of adjoining brain tissue (Figure 4A).

**FIGURE 5:**
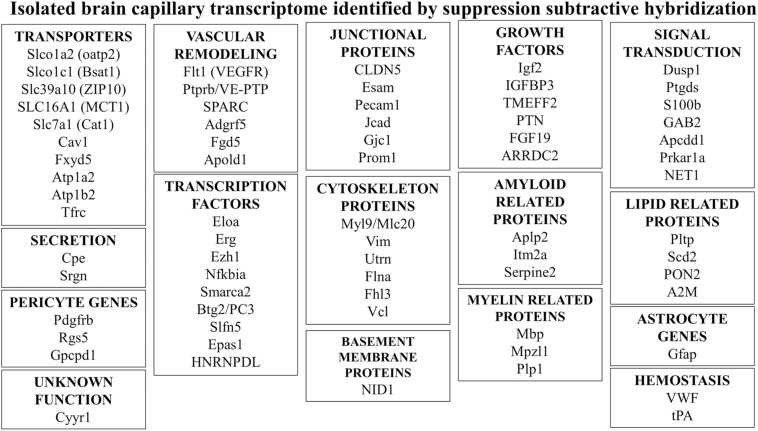
Genes of common function were identified by suppression subtractive hybridization following isolation of RNA isolated from microvessels obtained from either fresh rat brain or fresh human brain removed at neurosurgery (Li et al., 2001a, 2002; Shusta et al., 2002a). Additional details for all genes shown are given in Supplementary Table 1.

Poly(A) + RNA was isolated from either human brain microvessels (Figure 4A), or rat brain microvessels, to produce tester cDNA, and RNA was isolated from either autopsy human liver and kidney, or fresh rat liver and kidney to produce driver cDNA. This cDNA was generated from the isolated RNA by reverse transcription. The brain microvascular-enriched cDNA PCR products were purified and subcloned into pCR-2.1, followed by transformation of XL-1 Blue-competent *Escherichia coli.* The cDNA library of subtracted cDNA products typically yielded 10^6^ clones. Colonies were selected to inoculate 96-well plates. After growing for 2 days at 37°C, the cultures were blotted to a nitrocellulose filter, and standard Southern blotting was used to probe the membranes for either the subtracted or unsubtracted tester cDNA populations (Figures 4B,C). Clones selectively expressed in the subtracted tester cDNA are highlighted in Figure 4B. The cDNA from these clones was subjected to bidirectional DNA sequencing of the inserts, and the sequence compared to the GenBank database. A total of 70 genes, and 1 pseudogene, selectively expressed at the rat and human brain capillary were identified, and these genes are listed in Supplementary Table 1. In many cases, the original accession number has been updated, and genes that were originally identified as either expressed sequence tags (ESTs) or novel genes are now classified as known genes (Supplementary Table 1). These brain capillary enriched genes are grouped by common function in Figure 5. The isolated brain capillary is a multi-cellular structure and capillary-derived RNA includes transcripts for GFAP, an astrocyte-specific gene (Boulay et al., 2017), transcripts for Pdgfrb, Rgs5, and Gpcpd1, which are pericyte-specific genes (Chasseigneaux et al., 2018), and RNA encoding Mbp, Mpzl1, and Plp2, which are oligodendrocyte-related genes (Dugas et al., 2006; Ishii et al., 2009).

An advantage of the SSH approach to brain capillary genomics is that partial cDNAs are isolated for each gene, and these partial cDNAs can be used to screen brain capillary cDNA libraries to isolate the full length cDNA for any novel gene that is identified. The availability of the full length cDNA then allows for expression cloning of the gene and for a functional analysis of the gene. This was done in the case of a clone that was originally identified as K2 or LKH17 (Li et al., 2001a, 2002). Sequencing of the full length 2.6 kb cDNA showed this clone was a novel gene with a sequence distantly related to a liver specific anion transporter, and this gene was named BBB specific anion transporter type 1 (BSAT1). The gene was subsequently named organic anion transporting polypeptide 14 (Oatp14), or organic anion transporting polypeptide 1C1 (Oatp1c1), and is now classified as Slco1c1 (Supplementary Table 1). BSAT1/Slco1c1 was shown to be a transporter for thyroxine (T4), a thyroid hormone, and steroid hormone conjugates such as estradiol glucuronate (E2G) (Sugiyama et al., 2003; Tohyama et al., 2004). Slco1c1 has been cited as the principal BBB transporter mediating the influx of T4 from blood to brain (Lang et al., 2011). However, the *in vivo* BBB transport of thyroid hormones, T4 or triiodothyronine (T3), and sex steroid conjugates such as E2G, are inconsistent with the model that Slco1c1 mediates transport of these ligands at the BBB in the blood to brain direction. The transport of T3 and T4 across the BBB have been measured *in vivo* and T3 influx from brain to blood is faster than T4 influx (Pardridge, 1979); conversely, Slco1c1 has a low affinity for T3 (Chu et al., 2008). Sex steroid conjugates, such as E2G, which has a high affinity for Slco1c1 (Sugiyama et al., 2003; Chu et al., 2008), do not cross the BBB *in vivo* in the blood to brain direction (Pardridge et al., 1988). The principal BBB thyroid hormone transporter appears to be monocarboxylic acid transporter type 8 (MCT8), also known as SLC16A2, which has high affinity for both T3 and T4 (Friesema et al., 2003), and is expressed at the human brain capillary (Shawahna et al., 2011).

The rat brain capillary transcriptome was investigated by Enerson and Drewes (2006) using RNA isolated from rat brain capillaries and serial analysis of gene expression (SAGE). This approach does not use a subtraction step, and does not produce partial cDNAs. However, the relative expression of a given gene at the brain capillary is measured by SAGE, which gives a relative tag count that correlates with mRNA expression as measured by Northern blot analysis (Enerson and Drewes, 2006). Many brain capillary genes identified by SSH are also identified by SAGE, including Mbp, Cldn5, Esam, Cat1 (Slc7a1), BSAT1 (Slco1c1), and TfRc (TfR1) (Enerson and Drewes, 2006). The BBB transporters with the highest tag count included Glut1 (Slc2a1), MCT6 (Slc16a6), Cat1 (Slc7a1), and Bsat1 (Slco1c1) (Enerson and Drewes, 2006).

The identification of brain capillary endothelial cell specific transcripts was enabled by the use of fluorescent activated cell sorting (FACS) (Daneman et al., 2010). A transgenic mouse was engineered where the green fluorescent protein (GFP) gene was placed under the influence of the Tie2-endothelial cell specific promoter. The brains of the Tie2GFP transgenic mice were enzymatically homogenized, and the fluorescent brain endothelial cells were isolated by FACS prior to RNA isolation and hybridization to an Affymetrix mouse genome 430 2.0 gene chip. Pericytes adhered to endothelial cells were depleted with an antibody to platelet derived growth factor receptor beta (Pdgfrb), a pericyte specific protein (Figure 5). The top 4 genes that were >1000-fold enriched in brain endothelial cells, compared to liver endothelial cells, included inter-alpha trypsin inhibitory heavy chain 5 (Itih5), adenomatosis polyposis coli down-regulated 1 (Apcdd1), P-glycoprotein (Abcb1a), and Bsat1/Oatp1c1 (Slco1c1). Both Apcdd1 and Slc01c1 genes were identified as being highly enriched at the BBB by SSH (Supplementary Table 1). P-glycoprotein, other than Glut1, is one of the most enriched transporter proteins at the BBB (Table 1), which in the mouse is encoded by 2 genes, Abcb1a and Abcb1b (Kalabis et al., 2005).

The FACS-based approach to brain capillary endothelial genomics in health and brain disease was recently extended using a transgenic mouse expressing tamoxifen-inducible Cre-recombinase (Cre-ERT2) under the regulation of the vascular endothelial cadherin promoter (VECad) (Monvoisin et al., 2006), and expressing the tdTomato orange fluorescent protein following a week of tomoxifen induction (Munji et al., 2019). Brain endothelial cell gene expression was assessed in multiple disease models including kainic acid-induced seizures, permanent middle cerebral artery occlusion, experimental allergic encephalomyelitis, and traumatic brain injury (Munji et al., 2019). Gene expression in brain endothelial cells under these models was compared to gene expression in endothelial cells of peripheral organs. Genes selectively expressed in brain endothelial cells included genes identified by SSH (Supplementary Table 1) including transporters [Slc39A10 (a zinc transporter, ZIP10), Slco1c1 (BSAT1/Oatp1c1), and Slc7a1 (Cat1)], receptors (Tfrc, the transferrin receptor, Flt1, the vascular endothelial growth factor receptor), junctional proteins (Prom1), amyloid related genes (Itm2a), pericyte markers (Gpcpd1), lipid related proteins (Pltp), and signal transduction proteins (Apcdd1).

Magnetic activated cell sorting (MACS) of brain capillary cells has recently been used to identify the brain capillary pericyte-specific and smooth muscle-specific transciptomes (Chasseigneaux et al., 2018). Rat brain was enzymatically digested and endothelial cells were selected with an antibody against CD31 (Pecam1); microglia were selected with an antibody against CD11b; astrocytes were selected with an antibody against glutamate/aspartate transporter (GLAST), which is identical to EAAT1 (Slc1a3); oligodendrocytes were selected with an antibody to the O4 forkhead box protein (Fox04). Pericytes were isolated by negative cell sorting after selection of non-pericyte vascular cells (Chasseigneaux et al., 2018). Over 12,000 genes were cataloged following RNA sequencing (RNA Seq). Vascular smooth muscle cell specific genes included alpha-actin (Acta2); pericyte specific genes included Rgs5 and Pdgfrb; endothelial cell specific genes included P-glycoprotein (Abcb1) and claudin-5 (Cldn5); astrocyte specific genes included Gfap and aquaporin-4 (Aqp4).

Brain microvascular genomics allows for identification of genes selectively expressed in brain capillary endothelium, as well as other brain microvascular cells. The identification of transporter genes that are selectively expressed in the capillary endothelium provides the basis for “pharmaco-genomics” as a parallel platform for the pharmaco-proteomics platform produced by the QTAP efforts described above. However, once these transporters are identified, through either a genomics or proteomics approach, it is important to extend the work to a functional analysis of the transporters identified at the BBB. This is possible with expression cloning methods where the BBB transporter is expressed in either frog oocytes or mammalian cells so that the functional properties of the BBB transporter may be elucidated.

## Expression Cloning of Bbb-Specific Genes

**FIGURE 6:**
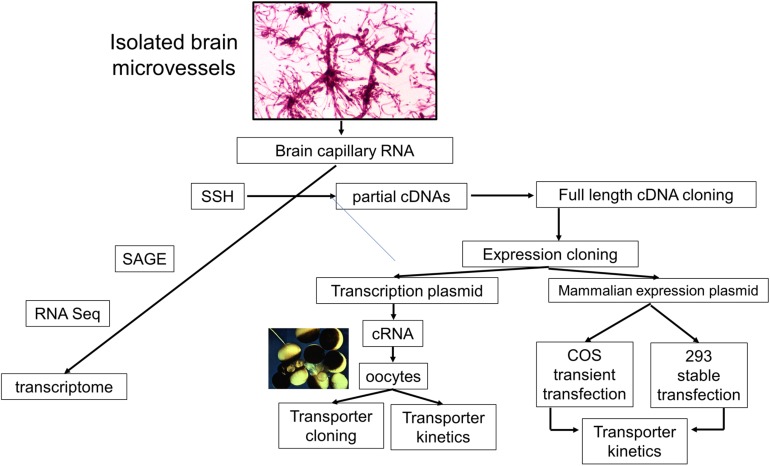
Pathways of investigation of brain microvascular genomics studies starts with the isolation of brain microvessels, and then microvessel-derived RNA. The brain capillary transcriptome may then be identified with suppression subtractive hybridization (SSH), serial analysis of gene expression (SAGE), or RNA sequencing (RNA Seq). The SSH methodology produces partial cDNAs, which can then be used to screen brain microvascular cDNA libraries for cloning of the full-length cDNA in either a transcription plasmid, for *in vitro* transcription of cloned RNA (cRNA), or a mammalian expression plasmid DNA for transient transfection of COS cells or stable transfection of human 293 cells. These expression cloning methods can be used to characterize the specific biochemical function, e.g., transporter kinetics, of genes found to be enriched in isolated brain microvessels. The microvessels shown were isolated from fresh rat brain. Adapted with permission from Pardridge (2005).

The identification of a CMT or RMT transporter at the BBB following either a brain capillary proteomics or brain capillary genomics investigation can be followed-up by clarification of the specific BBB transport functions mediated by the discovered transporter. This is enabled by expression cloning of the transporter and a functional analysis of the expressed transporter. One advantage of a SSH-based genomics program is that clones encoding partial cDNAs can be used to clone full length cDNAs (Figure 6). The full length cDNA might encode the full length expression cassette of the gene including the 5’- and 3’-untranslated region (UTR), or may only encode the open reading frame without the native 5’- or 3’-UTR. The full length cDNA may be subcloned into a transcription plasmid, such as pSPORT, to enable *in vitro* transcription of complementary RNA (cRNA) encoding the transporter. Such cRNAs may then be injected in frog oocytes for expression of the cloned transporter at the oocyte plasma membrane (Boado et al., 1999). Alternatively, the full length cDNA may be subcloned in a mammalian expression plasmid DNA followed by either transient expression in COS cells (Shusta et al., 2002b) or stable expression in human 293 cells (Chu et al., 2008).

### Expression of BBB Transporters in Frog Oocytes

Large neutral amino acid (LNAA) transport across the BBB is mediated via the LNAA transporter type 1 (Lat1, Slc7a5) (Boado et al., 1999). Lat1 forms a hetero-dimer with 4F2hc (CD98, Slc3a2) (Kanai et al., 1998). Co-injection of LAT1 and 4F2hc cRNAs into frog oocytes showed that tryptophan (Trp) transport was inhibited by other LNAAs, but not by either acidic amino acids (AA), basic AA, or sodium-free medium. A synthetic amino acid, 2-aminocyclo [2.2.1] heptane-2- carboxylic acid (BCH), which is specific for the leucine (L)-preferring amino acid transporter, inhibited Trp transport, but an analog, N-methylaminoisobutyric acid (NMAIB), which is specific for the alanine (A)-preferring amino acid transporter, had no effect of Trp transport into LAT1 cRNA-injected oocytes (Boado et al., 1999). The Km of Trp transport in oocytes was 31.5 ± 5.5 uM, which compares with the Km of Trp transport across the BBB *in vivo* (Pardridge, 1998).

The frog oocyte expression system, combined with site-directed mutagenesis (SDM), can be used to assess BBB transporter structure−activity relationships. This was exemplified by a study that used SDM to convert all 12 phylogenetically conserved cysteine (Cys) residues in the LAT1 transporter to serine (Ser) residues. The study was prompted by early work showing that BBB large neutral amino acid transport *in vivo* was selectively blocked by inorganic mercury, as compared to BBB transport of glucose (Pardridge, 1976). Inorganic mercury inactivates transporters by targeting Cys residues. The Ki of HgCl_2_ inhibition of phenylalanine (Phe) transport into LAT1 injected oocytes was 0.56 ± 0.11 uM (Boado et al., 2005). Mutation of Cys-160, which is part of an extracellular loop between transmembrane regions (TMR) 3 and 4, and which forms a disulfide with 4F2hc, did not alter LAT1 transport activity. However, mutation of Cys-439, which lies within TMR 11, completely blocked LAT1 transporter function (Boado et al., 2005).

Purine nucleosides, such as adenosine, cross the BBB *in vivo* via carrier-mediated transport, although there is minimal BBB transport of pyrimidine nucleosides, such as thymidine (Cornford and Oldendorf, 1975). Nucleosides are transported by either concentrative nucleoside transporters (CNT), such as CNT1 (Slc28a1), which prefers pyrimidine nucleosides, or CNT2 (Slc28a2), which prefers purine nucleosides, or equilibrative nucleoside transporters (ENT), such as ENT1 (Slc29a1) or ENT2 (Slc29A2), which transport both purine and pyrimidine nucleosides. CNT transporters are sodium-dependent and ENT transporters are sodium-independent (Young, 2016; Boswell-Casteel and Hays, 2017). ENT1 is expressed at the human and mouse brain capillary (Uchida et al., 2011). However, BBB transport of adenosine *in vivo* is sodium-dependent in the blood to brain direction (Pardridge et al., 1994), which suggests that CNT2 mediates BBB adenosine transport. This was confirmed by expression cloning in frog oocytes of the rat BBB adenosine transporter (Li et al., 2001b). An adenosine transporter was cloned, and sequencing showed this transporter to be CNT2 (Slc28a2). The Km of adenosine transport into frog oocytes injected with the CNT2 cRNA was 23.1 ± 3.7 uM, and adenosine transport was sodium dependent with a sodium K_0_._5_ of 2.4 ± 0.1 mM and a Hill coefficient of 1.06 ± 0.07 (Li et al., 2001b). The sodium K_0_._5_ of 2.4 mM is < 2% of the NaCl concentration in plasma, 140 mM, which indicates CNT2 is completely saturated by endogenous NaCl. Adenosine transport across the BBB *in vivo* is sodium-dependent (Pardridge et al., 1994), which excludes either ENT1 or ENT2 as a likely transporter of purine nucleosides, at least on the luminal membrane of the brain capillary endothelium.

Expression cloning of organic anion transporter Oat3 (Slc22a8) in frog oocytes demonstrated this transporter as the likely mediator of BBB efflux of organic anions, such as homovanillic acid (HVA), which is the principal metabolite of dopamine. HVA undergoes selective transport across the BBB in the brain to blood direction (Mori et al., 2003). Injection of Oat3 cRNA in frog oocytes identified the type of organic anions that compete for HVA transport via Oat3, and these include drugs, such as benzylpenicillin, and sex steroid conjugates, such as estrone sulfate (Mori et al., 2003).

The alanine serine cysteine transporters include Asct1 (Slc1a4) and Asct2 (Slc1a5). L-aspartic acid (Asp), an acidic amino acid and an excitatory neurotransmitter, undergoes selective efflux in the brain to blood direction across the BBB (Tetsuka et al., 2003), and L-Asp is transported into frog oocytes injected with either Asct1 or Asct2 cRNA (Tetsuka et al., 2003). It is proposed that the major active efflux transporter for L-Asp at the BBB is Asct2, since Asct2 expression is much higher than is Asct1 in cultured brain endothelium (Tetsuka et al., 2003). Extrapolation of findings in cell culture models to the *in vivo* state is done with caution, as many BBB genes are down-regulated in culture (see below, *In vitro* BBB Models).

Cholesterol transport across the BBB in the blood to brain direction is minimal owing to the avid binding to low density lipoprotein (Jin et al., 2019). Therefore, cholesterol is synthesized *de novo* in brain (Iuliano et al., 2015). Cholesterol made in brain is exported to blood as hydroxy cholesterol metabolites (Iuliano et al., 2015). A major cholesterol metabolite exported from brain to blood is 24S-hydroxycholesterol (24S-OH-Chol), and 24S-OH-Chol is transported in the brain to blood direction with a T_1__/__2_ of 101 mins (Ohtsuki et al., 2007). Early work based on frog oocyte uptake of 24S-OH-Chol implicated organic anion transporting polypeptide 2 (oatp2, Slco1b1) as a mediator of 24S-OH-Chol efflux from brain (Ohtsuki et al., 2007). More recent work implicates the role of P-glycoprotein (Abcb1) in the brain efflux of 24S-OH-Chol (Saint-Pol et al., 2013).

### Expression of BBB Genes in Mammalian Cells

There are multiple mammalian expression plasmids, in which transgenes are placed under the influence of a promoter, such as the cytomegalovirus (CMV) promoter, and a 3’-UTR, such as that derived from the bovine growth hormone (BGH) polyA termination sequence. Following the isolation of brain capillary derived RNA, cDNA can be produced and inserted in the mammalian expression plasmid. The expression plasmid may contain genes that enable high transient expression in COS cells (Shusta et al., 2002b), or may contain separation expression cassettes that allow for selection of stably transfected mammalian cell lines, such as human 293 cells (Chu et al., 2008). Early work developed antibody-based expression cloning following the transient transfection of COS cells with brain capillary derived expression plasmid DNA (Shusta et al., 2002b). In parallel, a polyclonal antiserum was produced by immunization of rabbits with brain capillary derived proteins (Pardridge et al., 1990b). Such antisera are broadly reactive, but are made specific for antigens expressed within brain capillary endothelial cells following the adsorption of the antisera with acetone powders of peripheral organs such as liver and kidney (Pardridge et al., 1990b). Antibody-based screening of BBB COS cell cDNA libraries and the absorbed anti-BBB antiserum led to the isolation of a cDNA for the Lutheran glycoprotein and the CD46 protein at the brain capillary (Shusta et al., 2002b, c). The CD46 protein is a receptor for certain meningococcal bacteria, e.g., *Neisseria meningitidis* (Johansson et al., 2003), and for exosomes derived from cancer cell lines (Kuroda et al., 2019).

Multiplex expression cloning (MEC) of brain capillary genes employs an absorbed BBB membrane protein specific polyclonal antiserum (BMPSA) and fluorescent activated cell sorting (FACS) of immunopositive human 293 cells transfected with a BBB cDNA library subcloned in a mammalian expression plasmid DNA (Agarwal and Shusta, 2009). This approach led to the identification of selective expression at the brain capillary of over 25 different proteins (Agarwal et al., 2010). In parallel, a novel approach to BBB genomics was developed in which brain and peripheral tissues were labeled with fluorescein-conjugated *Griffonia* simplicifolia agglutinin-I-B4, a lectin specific for endothelial cells. The microvessels from brain and peripheral organs were isolated by laser capture microdissection (LCM) followed by capillary RNA isolation (Agarwal et al., 2010). Quantitative PCR determined the relative abundance of transcripts for over 40 genes at the brain capillary as compared to capillaries in peripheral organs. This work paralleled another study that used LCM of brain capillaries immune-labeled with an antibody against CD31 [platelet endothelial cell adhesion molecule (PECAM)] and non-quantitative LC-MS to identify numerous junctional proteins, transporters, and enzymes selectively expressed at the brain capillary (Lu et al., 2008).

Blood-brain barrier transporter genes may also be stably expressed in mammalian cells lines to examine transporter function. Early BBB genomics studies with SSH and rat brain capillaries led to the cloning of the cDNA for a novel organic anion transporter named Bsat1/Slco1c1 (Li et al., 2001a). Human 293 cells were stably transfected with the full length cDNA encoding Bsat1/Slco1c1, either with or without the 5’-UTR of the Slco1c1 transcript (Chu et al., 2008). This work allowed for the identification of cytoplasmic proteins that bind the 5’-UTR of the Slco1c1 full length transcript to suppress translation. Removal of the 5’-UTR from the open reading frame led to high expression of the Slco1c1 transporter in the stably transfected 293 cells. This work described the unusual and asymmetric transport properties of Slco1c1 for both thyroid hormones, thyroxine (T4) and triiodothyronine (T3), and estradiol glucuronide (E2G). T4 has a high affinity for the transporter with a Km of 0.72 ± 0.10 uM and a lower affinity for E2G, which has a Km of 6.1 ± 0.05 uM. Whereas T4 inhibited E2G transport with a Ki of 0.53 ± 0.09 uM, E2G did not inhibit T4 transport (Chu et al., 2008). T3 weakly inhibited T4 transport with a Ki of 50 ± 17 uM, but T3 was a potent inhibitor of E2G transport with a 10-fold lower Ki of 4.2 ± 0.7 uM. These observations suggest an asymmetric topology exists on the Slco1c1 transporter, where one site exclusively transports T4, and a second transport site is shared by T4, T3, and E2G (Chu et al., 2008). Support for asymmetric dual site transporters had recently been reported for the Zn^+2^ transporter, ZIP4/Slc39a4 (Zhang et al., 2020).

In summary, BBB proteomics or BBB genomics programs that identify novel transporters at the brain capillary are a starting point for subsequent investigations of transporter function. The transporter can be expressed in either frog oocytes or mammalian cells to characterize the transporter profile. The cellular localization of the transporter at the brain capillary ought to be identified, as a transporter expressed in isolated brain capillaries may originate in endothelial cells, pericytes, smooth muscle cells or astrocyte foot processes. Finally, if the transporter is a target for brain drug delivery, then it is necessary to confirm expression of the transporter at the blood side, or luminal membrane, of the brain capillary endothelium.

## *Ex vivo* Transport and Receptor Binding in Isolated Brain Microvessels

**FIGURE 7:**
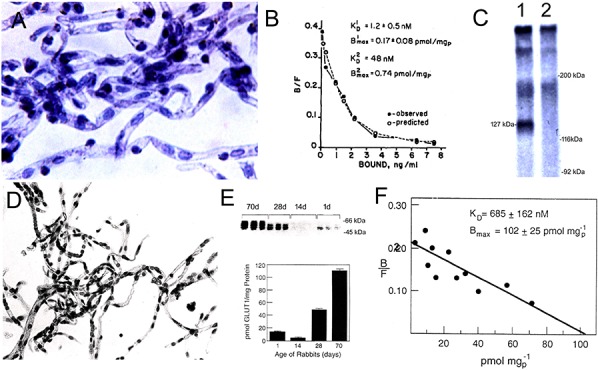
**(A)** Microvessels isolated from human autopsy brain are used as a source of the membrane bound human insulin receptor. Magnification is 200X. **(B)** Binding of [^125^I]-insulin to human brain microvessels is saturable, and a Scatchard plot of the binding data shows the dissociation constant (K_D_) of insulin binding to the HIR is high affinity with a K_D_ of 1.2 ± 0.5 nM. **(C)** Affinity cross-linking of [^125^I]-insulin to human brain microvessel-derived plasma membranes shows a single specific band of molecular weight of 127 kDa (lane 1), whereas this binding site is eliminated in the presence of 10 ug/mL unlabeled insulin (lane 2). Panels **(A–C)** are reprinted by permission from Pardridge et al. (1985a). **(D)** Microvessels isolated from adult rabbit brain are used for the characterization of the BBB glucose transporter. (**E**, top panel) Quantitative Western blot of microvessels isolated from rabbits 1, 14, 28, or 70 days of age, using a rabbit polyclonal antiserum against a carboxyl terminal 13 amino acid peptide of the human GLUT1 glucose transporter that is phylogenetically conserved. The 54 kDa Glut1 rabbit glucose transporter is detected, and varies with the postnatal age of the rabbit (**E**, bottom panel). Amount of Glut1 glucose transporter in microvessels isolated from rabbits of different ages is measured with the Quantitative western blot using purified human erythrocyte glucose transporter as the assay standard. **(F)** Scatchard plot of D-glucose displaceable binding of [^3^H]-cytochalasin B to brain microvessels isolated from the 70 day old rabbit shows the total number of all Glut binding sites in the rabbit brain microvessel is not significantly different from the total number of Glut1 sites as determined by quantitative Western blotting. Panels **(E,F)** are reprinted by permission from Dwyer and Pardridge (1993).

The CMT, RMT, or AET can be biochemically characterized in either freshly isolated or cryo-preserved microvessels derived from brain, including human autopsy brain. Figure 7A shows microvessels purified from human autopsy brain. These microvessels are metabolically impaired, but retain a structural integrity that enables biochemical characterization of RMT systems at the human BBB. The binding of insulin to the human insulin receptor (HIR) in human brain capillaries was saturable, as demonstrated with a radio-receptor assay (RRA) using [^125^I]-insulin (Pardridge et al., 1985a). Scatchard plots of the binding data showed insulin binds the HIR on the human BBB with high affinity and a dissociation constant (KD) of 1.2 ± 0.5 nM (Figure 7B). The insulin binding site on the human capillary was the classical HIR, as demonstrated by affinity cross-linking of [^125^I]-insulin to plasma membranes isolated from the brain capillary. The molecular weight (MW) of the [^125^I]-insulin binding site at the human BBB was 127 kDa (Figure 7C), which is identical to the MW of the alpha subunit of the peripheral HIR (McKern et al., 2006). There is a separate receptor, unrelated to the insulin receptor, on the human brain microvessel that binds with high affinity both insulin-like growth factor (IGF)-1 and IGF-2 (Duffy et al., 1988). Affinity cross-linking of [^125^I]-human IGF-2 to human brain microvessels shows the MW of the IGF-2 binding site is 141 kDa. These data indicate the cation independent mannose 6-phosphate receptor (CI-M6PR), which also has a high affinity for IGF-2, and is also called the IGF2 receptor (IGF2R), is not expressed at the human BBB as this receptor has a MW of 250 kDa (Olson et al., 2010). Isolated human brain microvessels have also been used analyze other RMT systems at the BBB, and to characterize the high affinity binding of transferrin (Tf) to the Tf receptor (TfR), and the high affinity binding of leptin to the leptin receptor, at the human BBB (Pardridge et al., 1987a; Golden et al., 1997).

Isolated brain microvessels can also be used to quantify the expression of CMT systems at the BBB. There are over 10 glucose transporters (Glut). While it is generally recognized that the Glut1 isoform (Slc2a1) is the major glucose transporter at the BBB, other Glut isoforms including Glut3 (Slc2a3) and the insulin-dependent Glut4 (Slc2a4) are said to function as glucose transporters at the BBB, based on studies in cultured endothelium (Al-Ahmad, 2017). The relative role of the Glut1 isoform at the BBB *in vivo* was examined with isolated rabbit brain microvessels, which are shown in Figure 7D. The expression of the immunoreactive Glut1 in these microvessels was measured with a quantitative Western blot using purified human erythrocyte GLUT1 as an assay standard (Figure 7E). The quantitative Western blot showed the level of Glut1 expression in brain microvessels in mature 70 day old rabbits was 111 ± 3 pmol per mg protein (Dwyer and Pardridge, 1993), which compares well with the level of Glut1 determined in isolated brain microvessels with QTAP (Table 1). The total number of glucose transporters in the microvessels was measured by quantitation of D-glucose displaceable cytochalasin B binding sites; Scatchard analysis of these binding data showed the total number of D-glucose displaceable binding sites on the capillary was 102 ± 25 pmol per mg protein (Figure 7F). Therefore, >95% of glucose transporters expressed at the BBB are represented by the Glut1 isoform. There is extensive developmental regulation of Glut1 at the BBB, and this transporter is down-regulated in post-natal animals (Figure 7E). The level of Glut1 in microvessels was measured with the quantitative Western blot, and brain microvessel Glut1 levels isolated from 1, 14, 28, and 70 day rabbits is 13 ± 2, 4 ± 1, 49 ± 2, and 111 ± 3, respectively (Dwyer and Pardridge, 1993). This post-natal suppression of the BBB Glut1 during the suckling period parallels the preferential oxidation of ketone bodies in brain in the post-natal period (Cotter et al., 2011).

Isolated brain microvessels can also be used to assess *ex vivo* transport of solutes or drugs via CMT or AET systems (Chan and Cannon, 2017). Microvessels isolated from autopsy human brain were used to characterize the Michaelis-Menten kinetics of uptake of large neutral amino acids (Hargreaves and Pardridge, 1988). Phenylalanine uptake by the microvessels, which had a Km of 0.30 ± 0.08 uM, was >90% mediated by the leucine (L)-preferring system, now known as Lat1 (Slc7a5). However, leucine uptake, which had an Km of 3.3 ± 0.7 uM was mediated by several transporters, including the L-system, the alanine-serine-cysteine (ASC)-preferring system, now known as Slc7a10, and the alanine (A)-preferring system, now known at Ata2 (Slc38a2). Uptake of amino acids by isolated capillaries is a combination of simultaneous transport across both the luminal and abluminal endothelial membranes, because the microvessels are patent, as demonstrated with 0.5 micron polystyrene microspheres (Hjelle et al., 1978). Nevertheless, there is reasonable correlation between the Km values of large neutral amino acid uptake into isolated human brain microvessels and the Km values of large neutral amino acid influx from blood to brain across the rat BBB *in vivo* (Table 2). The Km values for phenylalanine and tyrosine are higher *in vivo*, but the Km values *in vivo* in the rat for the other 6 large neutral amino acids in Table 2 are closely approximated by the Km values *in vitro* for the human brain microvessel.

**TABLE 2:** Comparison of large neutral amino acid Km values in isolated human brain capillary *in vitro* and rat blood-brain barrier *in vivo*.

**Neutral amino acid**	**Km (uM) Human brain microvessel**	**Km (uM) Rat BBB *in vivo***
Phenylalanine	0.30 ± 0.08	6.9 ± 0.6
Tyrosine	1.3 ± 0.4	16.1 ± 1.1
Isoleucine	2.7 ± 1.1	10.0 ± 0.7
Tryptophan	3.0 ± 0.7	9.1 ± 0.8
Leucine	3.3 ± 0.7	9.9 ± 0.9
Methionine	5.1 ± 2.5	4.2 ± 1.0
Histidine	5.1 ± 2.0	10.2 ± 0.4
Valine	8.8 ± 4.6	8.2 ± 0.5

*Human brain microvessel Km values *in vitro* from Hargreaves and Pardridge (1988); rat BBB Km values *in vivo* from Smith et al. (1987).*

So as to separate amino acid transport at the luminal and abluminal endothelial membranes, methods were developed that involved Ficoll gradient separation of these membranes (Betz et al., 1980). This method was then used to characterize amino acid transport into the vesicles formed by the separated luminal and abluminal membranes (Sanchez del Pino et al., 1992; Hawkins et al., 2006). Phenylalanine transport into the luminal membranes vesicles was sodium independent consistent with uptake via the L-system or Lat1. Amino acid transport into abluminal membrane vesicles was sodium dependent and blocked by the A-system specific inhibitor, NMAIB (Sanchez del Pino et al., 1992), which localized the A- system (Ata2) to the abluminal membrane. These findings were confirmed by QTAP measurements of CMT transporters in luminal and abluminal capillary membrane vesicles, which showed the Ata2 transporter was only expressed on the abluminal membrane (Kubo et al., 2015). Transport measurements with brain capillary derived abluminal vesicles showed the ASC transporter (Slc7a10), the glutamine (N)-preferring transporter, now known as the sodium-coupled neutral amino acid transporter (Snat1/2, Slc38a1/2) (Yamada et al., 2019), the excitatory amino acid transporters EAAT1 (Slc1a3), EAAT2 (Slc1a2), and EAAT3 (Slc1a1), are selectively expressed at the abluminal membrane (Hawkins et al., 2006). The sodium dependent taurine transporter (Taut, Slc6a6) is selectively expressed at abluminal membrane vesicles isolated from brain capillaries (Rasgado-Flores et al., 2012). SLC gene family transporters selectively expressed at the abluminal membrane of the capillary endothelium may mediate the preferential efflux of metabolites in the brain to blood direction. Such active efflux transporters (AET) also include ATP binding cassette (ABC) gene family transporters.

Isolated brain microvessels may also be used to assess *ex vivo* transport of ligands transported via ABC transporters, such as P-glycoprotein (Pgp, Abcb1), breast cancer resistance protein (Bcrp, Abcg2), and the multi-drug resistance protein-2 (Mrp2, Abcc2). The chemotherapeutic agent, paclitaxel, is a Pgp substrate and is excluded from entry into brain. The uptake of fluorescent analogs of paclitaxel by isolated brain microvessels was measured by fluorescent microscopy, and this uptake was inhibited by Pgp-blockers such as valspodar (Fellner et al., 2002). The activity of the ABC transporters at the BBB are subject to regulation via signal transduction pathways (see below, Signal Transduction).

## Signal Transduction in Isolated Brain Microvessels

Cellular function is mediated by a diversity of signal transduction pathways, which invariably are linked to phosphorylation and de-phosphorylation of specific proteins, and such processes play a major role in neurotransmission and synaptic plasticity (Woolfrey and Dell’Acqua, 2015). With the exception of the testis, the brain has nearly a 10-fold greater number of tissue-specific phosphoproteins among the organs of the body (Lundby et al., 2012). Therefore, it was surprising to find that the level of protein phosphorylation and de-phosphorylation in isolated brain capillaries was as high as that found in capillary-free brain cell membranes (Pardridge et al., 1985b). Protein kinases are classified as either tyrosine kinases or serine/threonine kinases. Tyrosine kinases are typically linked to cell membrane receptors, such as the insulin receptor (Insr) or platelet derived growth factor receptor (Pdgfr), and are activated following ligand binding. Serine/threonine kinases are activated by signal transduction pathways involving cyclic AMP (protein kinase A), cyclic GMP (protein kinase G), calcium/calmodulin (protein kinase CaMK), or diacylglycerol (protein kinase C). The activity of all 4 of these classes of serine/threonine kinases were identified in isolated rat brain capillaries (Olah et al., 1988; Edgardo Catalan et al., 1996). There is a need for further investigation of the regulation of signal transduction pathways at the brain microvessel, including the regulation of G proteins and the related accessory proteins (Sato et al., 2006).

Signal transduction pathways at the brain microvasculature have been studied primarily in the case of modulation of BBB permeability through modifications of tight junction proteins, or regulation of endothelial transporter activity, particularly in the case of the ABC transporters, such as P-glycoprotein. In the case of endothelial tight junctions:

• Bauer et al. (2014) reviews the role of more than a dozen transmembrane junctional proteins including the claudins, occludins, and the zonula occludens (ZO). Protein phosphorylation of these tight junction proteins is a major regulatory mechanism for tight junction assembly and maintenance in health and disease.
• In an experimental model of cerebral embolism in the rat, brain capillaries were isolated and protein phosphorylation of occludin and ZO-1 was examined. Occludin tyrosine-phosphorylation was enhanced by the cerebral embolism, and this was associated with an increase in activity of the c-Src tyrosine kinase, and a decrease in the level of endothelial occludin (Kago et al., 2006). Reduced junctional protein expression may lead to BBB disruption following the embolism.
• Following induction of reversible cerebral ischemia in the rat, capillaries were isolated, and enhanced tyrosine phosphorylation of endothelial occludin was demonstrated, which was associated with BBB disruption following cerebral ischemia (Takenaga et al., 2009). The administration of 4-amino-5-(4-chlorophenyl)-7-(t-butyl) pyrazolo [3,4-d]pyrimidine (PP2), a Src-family tyrosine kinase inhibitor, diminished the phosphorylation of occludin, and reduced the BBB disruption following cerebral ischemia (Takenaga et al., 2009).
• In an experimental allergic encephalomyelitis (EAE) model, where the BBB is disrupted, endothelial occludin de-phosphorylation was altered (Morgan et al., 2007), and such findings may be a model of BBB permeability changes in disease states such as multiple sclerosis.

Signal transduction pathway in isolated brain capillaries have been shown to alter the activity of ABC-type transporter systems in the brain endothelium, including Pgp (Abcb1), BCRP (Abcg2), and MRP2 (Abcc2):

• Exposure of isolated brain microvessels to the proinflammatory cytokine, tumor necrosis factor (TNF)- α, caused an increase in endothelin (ET)-1-related signaling, which was associated with a reduction in brain capillary Pgp function (Hartz et al., 2006). The role of TNFα in Pgp activity was mediated through protein kinase C (PKC)-dependent signaling in the brain capillary (Rigor et al., 2010). The TNFα effect on capillary Pgp was reduced by a PKC inhibitor, and replicated by a PKC activator; *in vivo* administration of the PKC activator caused an increase in the brain uptake of verapamil, a Pgp substrate. The study shows that PKC activation may alter Pgp activity at the BBB *in vivo* (Rigor et al., 2010).
• Vascular endothelial growth factor (VEGF) also inhibits brain capillary Pgp activity, and the effect was blocked by inhibitors of the VEGF receptor, Flk-1 and the Src kinase (Hawkins et al., 2010).
• Nuclear transcription factors such as erythroid-2 related factor-2 (Nrf2) have been implicated in the regulation of Pgp activity. The Nrf2 inhibitor, sulforaphane (SFN), was administered to rats, and brain capillaries were isolated (Wang et al., 2014). SFN administration caused an increase in brain microvessel activity of Pgp, BCRP, and MRP2. The effect of SFN on AET system activity at the brain capillary was not observed in Nrf2 knockout mice (Wang et al., 2014).
• Certain ABC transporters such as Mrp2 may be selectively regulated by glutamatergic receptors at brain capillaries. The addition of glutamate, an excitatory neurotransmitter, or N-methyl D-aspartic acid (NMDA), causes an increase in capillary Mrp2 activity (Luna-Munguia et al., 2015). The role of the NMDA receptor was confirmed by the observation that a NMDA receptor blocker, MK-801, inhibited the glutamate-mediated increase in Mrp2 activity in brain capillaries. More recent work suggests the glutamate enhancement of ABC transporter activity is mediated via cytosolic phospholipase A2 (cPLA2) (Hartz et al., 2019).
• The lipid sensor peroxisome proliferator-activated receptor alpha (PPARα), which is a major regulator of lipid metabolism, also regulates brain capillary ABC transporters, Pgp, BCRP, and Mrp2 (More et al., 2017). Isolated brain capillaries were exposed to clofibric acid (CFA), which is a PPARα agonist, and this treatment caused an increase in the activity of all 3 ABC transporters (More et al., 2017). CFA lowers plasma cholesterol and triglycerides, and is still prescribed in certain regions. It is possible that the administration of lipid lowering fibrates such as CFA may alter BBB permeability to certain pharmaceutical agents that are substrates for the ABC efflux systems expressed at the brain capillary.
• Zinc chloride (ZnCl_2_) is taken as a nutritional supplement, and recent work implicates the Zn^+2^ cation as an agent that increases ABC transporter activity in brain capillaries through an endothelin pathway that leads to activation of protein kinase C (Zaremba et al., 2019). The Zn^+2^ cation may gain access to brain from blood owing to the expression of Zip10 transporter (Slc39a10) at the brain microvasculature, which was identified by BBB genomics studies (Li et al., 2002; Daneman et al., 2010; Munji et al., 2019; Supplementary Table 1).
• The endothelial cytoskeleton plays a role in membrane localization of ABC transporters in brain capillaries and this pathway was investigated by testing the effects of members of the ERM family of proteins, ezrin (Ezn), radixin (Rdx), and moesin (Msn), which are required for maintenance of the cytoskeleton via crosslinking of actin filaments and membrane transporters (Hoshi et al., 2019). The effect of the ERM proteins on Pgp activity in the hCMEC/D3 line of human brain endothelium was investigated by siRNA knockdown of Ezn, Rdx, or Msn. ERM knockdown caused a reduction in Pgp and BCRP activity in the cultured cells. The expression of the ERM proteins in microvessels isolated from human autopsy brain was confirmed by QTAP measurement (Hoshi et al., 2019).

## Isolation of Microvessels From Human Brain in Neurologic Disease

The isolation of microvessels from human brain is a potentially powerful approach to investigation of the role of the microvasculature in human neurological disease. Microvessels have been isolated from non-frozen human autopsy brain (Pardridge et al., 1985a), from frozen human brain bank specimens (Pardridge et al., 1987b), and from fresh human brain taken at neurosurgery (Shusta et al., 2002a). Microvessels have been isolated from brains of human subjects with Alzheimer’s disease (AD), multiple sclerosis (MS), epilepsy, and brain cancer.

### Alzheimer’s Disease

**FIGURE 8:**
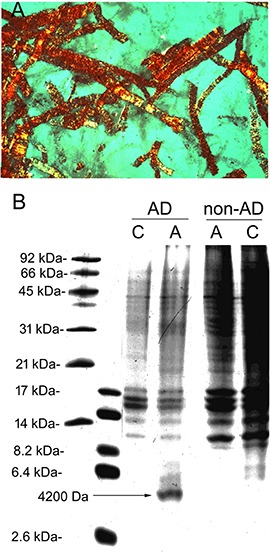
**(A)** Pre-capillary arterioles captured at the top of a 210 micron nylon mesh were isolated from human autopsy Alzheimer’s disease (AD) brain, cyto-centrifuged to a glass slide, stained with 0.05% Congo red, and visualized with polarized light. Magnification is 200X. The apple-green birefringence shows the presence of abundant amyloid in these AD brain microvessels. These arteriolar AD brain microvessels retained by a 210 micron nylon mesh are labeled “A” microvessels. The microvessels that passed through the 210 micron nylon mesh were largely capillaries and labeled as “C” microvessels. **(B)** A type and C type microvessels were isolated from both AD and non-AD human autopsy brain and solubilized in formic acid and separated on urea SDS–PAGE gels. A unique 4200 Da peptide is found in the arteriolar fraction of AD microvessels, not found in the same fraction from non-AD brain. Panels **(A,B)** are reprinted by permission from Pardridge et al. (1987c).

The isolation of brain microvessels from AD brain may contribute to the understanding of the pathogenesis of Aβ amyloid in AD, since Aβ amyloid plaques arise from the peri-microvasular surface of the brain in AD (Miyakawa, 2010). Microvessels were isolated from human AD autopsy brain, and the pre-capillary arteriole fraction was selectively enriched by extracting vessels trapped at the top of a 210 micron nylon mesh (Pardridge et al., 1987c). Staining of the AD cortical microvessels with Congo Red and visualization with polarized light showed the apple green birefringence typical of amyloid (Figure 8A). These vessels were solubilized in formic acid and the extract was analyzed by urea sodium dodecylsulfate polyacrylamide gel electrophoresis, followed by Coomasie blue staining/destaining (Figure 8B). The arterioles isolated from AD brain showed the presence of a 4200 Da peptide. This peptide isolated from cortical microvessels of AD brain had the same size as the Aβ peptide isolated from meningeal surface vessels of AD brain (Glenner and Wong, 1984). The 4200 Da peptide was extracted from the gel, and N-terminal amino acid sequencing confirmed this 4200 Da peptide isolated from cortical microvessels of AD brain was identical to the Aβ amyloid peptide found in meningeal vessels in AD (Glenner and Wong, 1984), and in neuritic plaque amyloid isolated from AD brain (Masters et al., 1985). Amino acid composition analysis of the cortical microvessel isolated from AD brain showed the presence of 1.0 threonine (Thr) residues. There are no Thr residues in Aβ^1–40^ or Aβ^1–42^, as the Thr residue is found at position 43 of Aβ^1–43^ (Masters et al., 1985). Subsequently, microvessels were isolated from AD brain, and the microvessel fraction analyzed was the filtrate obtained following passage through a 200 micron nylon mesh (Miller et al., 1993), a method that would exclude pre-capillary arterioles. The amyloid isolated from this fraction contained only 0.2 Thr residues per Aβ monomer, which suggests there is a heterogeneity of C-terminal truncated forms of Aβ in capillary and arteriolar segments of the AD microvasculature.

The Aβ amyloid peptide of AD is proteolytically derived from the amyloid peptide precursor (APP) (Masters et al., 1985). Beta-secretase 1 (BACE1) makes an internal cleavage of the APP to generate a 99 amino acid C-terminal fragment of APP, alternatively designated CT99 (Shi et al., 2003) or β-C-terminal fragment (CTF) (Kirabali et al., 2019). CT99 is then degraded by gamma-secretase to Aβ^1–39/43^ (Shi et al., 2003). These fragments may be further converted by gamma-secretase to Aβ^1–34^ (Shi et al., 2003). Aβ^1–34^ may play a role in the early pathogenesis of the microvascular amyloid angiopathy of AD, as this peptide is elevated in cerebrospinal fluid (CSF) in the early stages of AD (Kirabali et al., 2019). Microvessels were isolated from autopsy human brain with either AD or non-AD diagnoses, and the presence of Aβ^1–34^ in AD microvessels correlated with a pericyte marker, PDGFRβ (Kirabali et al., 2019). These findings suggest a role for the brain microvascular pericyte in the clearance of Aβ amyloid peptides in early AD. The other mural cell, the microvascular smooth muscle cell, may also play a role in the development of vascular amyloid in AD. Microvessels were isolated from double transgenic Tg-SweDI AD mice and C57Bl/6 control mice, and smooth muscle α-actin (SMA) Western blotting showed a 2-fold elevation in αSMA in AD microvessels (Hutter-Schmid and Humpel, 2016).

Microvessels have been isolated from human AD brain and such investigations have shown an increase in nitric oxide synthase (Dorheim et al., 1994), an increase in inflammatory cytokines such as interleukin-1β or TNFα (Grammas and Ovase, 2001), and an increase in vasoactive peptides such as endothelin (ET)-1 in AD brain microvessels (Luo and Grammas, 2010). Both TNFα and ET1 have been implicated in the development of amyloid in AD (Palmer et al., 2010; Chang et al., 2017).

A genomics analysis of human AD brain microvessels was reported by Wang et al. (2012). Microvessels were isolated from frozen brain bank specimens of either AD brain or control brain, and were purified by filtration through a 210 micron nylon mesh with collection at the top of a 53 micron nylon mesh, which selectively purifies capillaries from human brain. The yield of capillaries was 6-10 mg capillary protein per 15 grams of human cortex (Wang et al., 2012). Since brain is comprised of about 100 mg protein per gram brain (Dunlop et al., 1994), the brain capillary constitutes about 0.5% of total brain protein. The capillary derived RNA was hybridized to an RNA Agilent human 4 × 44K oligonucleotide chip for both the AD and non-AD specimens. The study showed altered expression of >2,000 genes in capillaries isolated from AD capillaries as compared to non-AD capillaries (Wang et al., 2012).

### Multiple Sclerosis

T-lymphocyte transport (diapedesis) through the BBB is a central event in the development of the brain lesions in MS (Prat et al., 2002). An early pathologic hallmark of MS is a peri-vascular cuffing of lymphocytes (Wu and Alvarez, 2011). Therefore, the isolation of microvessels from human MS brain may provide insight into the pathogenesis of this condition. Microvessels can be isolated from plaque tissue of frozen brain bank brain (Pardridge et al., 1987b). Microvessels isolated from human MS brain show a prominent expression of the class II HLA DR antigen (Pardridge et al., 1989; Washington et al., 1994), as shown in Figure 3E. Unfortunately, there have been few recent attempts to characterize the microvessels form human MS brain. This would be important to understanding the diapedesis of lymphocytes through the BBB in MS (Risau et al., 1990; Wimmer et al., 2019). Certain cell adhesion molecules, such as platelet endothelial cell adhesion molecule-1 (PECAM1, CD31) may play a role in lymphocyte trafficking to brain (Wimmer et al., 2019). An experimental model of MS is experimental allergic encephalomyelitis (EAE). Encephalitogenic T-lymphocytes penetrate the spinal cord BBB in EAE, and these pathways can be monitored *in vivo* with two-photon microscopy adapted to a spinal cord window (Haghayegh Jahromi et al., 2017).

### Epilepsy

A major problem in the treatment of human epilepsy is drug resistance to anti-convulsants. Several anti-convulsant agents are substrates of P-glycprotein (Pgp, Abcb1). As discussed above in the Signal Transduction section, brain capillary endothelial PgP is subject to up- and down-regulation through a variety of signal transduction pathways active at the brain microvasculature. An up-regulation of Pgp at the BBB would cause resistance to those anti-convulsants that are Pgp substrates. Such an up-regulation of Pgp in both experimental epilepsy and human epilepsy has been demonstrated (Hartz et al., 2019). In either experimental or human epilepsy, brain microvessels were isolated and analyzed by a semi-quantitative Western blot procedure using capillary electrophoresis and the C219 monoclonal antibody, which reacts against either rat or human Pgp (Hartz et al., 2019). Additional work on the isolation of microvessels from experimental or human epilepsy is warranted. As shown in Figure 5, a number of genes involved in vascular remodeling have been identified in brain microvascular genomics programs. Marchi and Lerner-Natoli (2013) review the role of vascular remodeling and angiogenesis in the foci of brain that trigger human epilepsy. Such studies suggest the NVU plays a role in the pathogenesis of epilepsy, and an understanding of this role could be enhanced by the investigation of the molecular differences in microvessels isolated from control and human epileptic brain.

### Human Brain Cancer

The brain microvasculature plays a role in the development of human brain cancer, as the growth of the cancer is limited, in part, by angiogenesis, as demonstrated by intravital imaging of the microvasculature in experimental brain cancer (Fukumura et al., 2010). One study characterizes brain microvessels isolated from experimental brain tumors in rats (Silbergeld and Ali-Osman, 1991). Unfortunately, there have been few attempts to characterize the microvessels isolated from human brain cancer. Brain cancer is the one human neurologic condition where there is an abundance of fresh tissue removed at neurosurgery. The isolation of microvessels from human brain cancer, including glioblastoma, astrocytoma, oliogodendroglioma, or meningioma was reported by Magnoni et al. (1988). This study was focused on the expression of beta-adrenergic receptors at the tumor microvasculature, and such receptors were not detected in microvessels isolated from glial tumors, although the receptors were expressed in microvessels in meningioma type brain tumors (Magnoni et al., 1988). Given the importance of the blood-tumor barrier in the pathogenesis and treatment of human brain cancer, it is surprising that there are not additional investigations of mirovessels isolated from human brain cancer removed at neurosurgery. Microvessels were isolated from human brain tumors, but then these were used to produced cultured endothelial cells (Unger et al., 2002). As discussed in the next section, cultured brain endothelium do not replicate all of the properties of brain capillary endothelium *in vivo*, as there is down-regulation of brain endothelial gene expression when these cells are grown in cell culture.

## *In vitro* Models Of Brain Endothelium Grown In Cell Culture

### History of *in vitro* BBB Model

Attempts to develop an “*in vitro* BBB” model with brain endothelial cells grown in cell culture date back over 30 years (Bowman et al., 1983). However, cultured endothelium lost many BBB properties *in vitro*, and this was attributed to the loss of astrocyte foot process induction of BBB properties (DeBault and Cancilla, 1980). Dehouck et al. (1990) developed an endothelial cell/astrocyte co-culture system, where primary cultures of bovine brain endothelial cells (BBCE) were grown on one side of a Millicell-CM culture plate insert, and newborn rat brain astrocytes were grown on the opposite side of the insert. The pore size of the insert was 0.4 microns. Focal increases in BBB related gene products, such as gamma glutamyl transpeptidase (GGTP), in endothelial cells were observed with the co-culture system, and this was attributed to the presence of diffusible factors in astrocyte conditioned medium. This small pore “Trans-well” model has been used as the principal *in vitro* BBB model for many years, despite the early findings of Meyer et al. (1991) and Hurwitz et al. (1993), which indicated the crucial role of direct contact of the endothelium by astrocyte processes. The induction of BBB properties in porcine brain endothelial cells by newborn rat brain astrocytes were not observed in a co-culture, but required the cell to cell contact found with mixed cell cultures (Meyer et al., 1991). Induction of BBB properties with a co-culture system was possible, but only if the pore size of the insert was 3.0 microns, which allowed for cell to cell contact (Hurwitz et al., 1993). In this work, the co-culture system employed the growth of human fetal brain astrocytes on the under-side of the culture plate insert, and human umbilical endothelial cell (HUEC) on the opposing side of the insert. If the pore size of the insert was 3.0 microns, then astrocyte foot process could extend through the large pore and come in direct contact with the endothelial cells, and induction of BBB-related gene products, such as the Glut1 glucose transporter or GGTP, was observed (Hurwitz et al., 1993). However, if the pore size was 0.45 microns, then astrocyte foot processes could not extend through this small pore, nor come in contact with the endothelial cells, and no induction of BBB properties was observed (Hurwitz et al., 1993). Despite these findings, the pore size of Trans-well filters used for *in vitro* BBB models is typically only 0.4 microns (Santaguida et al., 2006; Canfield et al., 2017). The use of a Transwell with a 0.4 micron pore size assumes that the induction of BBB properties in co-culture is mediated by a soluble, diffusible factor (Cecchelli et al., 2014). Conversely, the findings of Meyer et al. (1991) and Hurwitz et al. (1993) suggest that induction of BBB properties requires direct cell to cell contact. The cell−contact model of BBB induction is consistent with the very close relationship between endothelial cells and astrocyte foot processes within the NVU (Figure 2A), and these cells are separated by a distance of only 20 nm (Mathiisen et al., 2010). A mixed cell culture system, also called a spheroid system, was developed by Urich et al. (2013), where a human brain endothelial cell line, a human pericyte cell line, and human astrocyte cell line were grown in a mixed culture. Spheroids were formed in a droplet placed in a well of a 3-dimensional hanging drop culture plate. Cho et al. (2017) has proposed the use of the spheroid mixed culture as an *in vitro* BBB model for screening drug transport. The development of an *in vitro* BBB model for high throughput screening of BBB drug transport has been long sought by the pharmaceutical industry (Reichel, 2006). However, the use of an *in vitro* BBB model assumes this model faithfully replicates the permeability properties of the BBB *in vivo*. *In vivo*/*in vitro* BBB comparisons are generally of 3 types: (a) tight junction formation as reflected in trans-endothelial electrical resistance (TEER) measurements, (b) BBB permeability properties as reflected in the permeability coefficient (Pe) for a poorly diffusible compound such as sucrose, and (c) BBB-tissue specific gene expression in cell culture.

### Trans-Endothelial Electrical Resistance With *in vitro* BBB Models

The TEER of pial vessels on the surface of the brain is about 1500 ohm⋅cm^2^ (Butt et al., 1990). However, pial vessels do not exhibit complete BBB properties, as these vessels have only a glial limitans, and are not representative of intra-parenchymal vessels (Allt and Lawrenson, 1997). The TEER at intra-parenchymal vessels in brain is estimated to be about 8,000 ohm⋅cm^2^ (Smith and Rapoport, 1986), which is 400-fold higher than the TEER across the leaky choroid plexus *in vivo*, 20 ohm⋅cm^2^ (Butt et al., 1990). The TEER across a monolayer of a widely used human brain endothelial cell line, the hCMEC/D3 line, is only <50 ohm⋅cm^2^ (Al-Ahmad, 2017). This is actually lower than the TEER in a monolayer of mouse 3T3 fibroblasts, 200 ohm⋅cm^2^ (Canfield et al., 2017). The TEER is increased to 800 ohm⋅cm^2^ in a co-culture model of primary mouse brain endothelial cells and newborn mouse brain astrocytes grown in a Transwell system with a 0.4 micron pore size (Coisne et al., 2005). The addition of pharmacological concentrations, e.g., 5-10 uM, of retinoic acid (RA) to endothelial cultures causes a marked increase in TEER to a level of 1700-3000 ohm⋅cm^2^ in lines of brain endothelial cells (Lippmann et al., 2014). RA is a biologically active metabolite of retinol (vitamin A), and binds to RA receptors to trigger signal transduction pathways that lead to an increase in cell expression of tight junction proteins, such as the claudins or ZO-1 in multiple epithelial cell lines (Telgenhoff et al., 2008; Kurti et al., 2013). In brain endothelial cell lines, such as the hCMEC/D3 line, a 5 uM concentration of RA increases tight junction proteins such as ZO-1, and gap junction proteins, such as VE-cadherin, via pathways involving the Wnt signaling-associated transcription factors (Mizee et al., 2013). RA increases the level of junctional protein mRNAs up to 20-fold in *in vitro* BBB models, including human pluripotent stem cells (hPSC), which can be differentiated to express certain BBB properties in cell culture (Qian et al., 2017). The RA-induced increase in TEER values with *in vitro* BBB models, therefore, correlates with the known induction of junctional protein expression by RA. The differentiated hPSC, in the presence of 10 uM RA, has been extended to a 0.4 micron pore size Transwell co-culture with astrocytes, pericytes, or neurons, in different combinations, where all cell types originated from the same human hPSC donor (Canfield et al., 2019). Human PSCs transfected with genes encoding Wnt signaling-related transcription factors have enhanced tight junction protein expression and increased resistance in cell culture models (Roudnicky et al., 2020).

### Sucrose Permeability Coefficients (Pe) With *in vitro* BBB Models

The Pe *in vivo* for BBB solute transport is derived from the permeability-surface (PS) product, and the brain capillary surface area (S), which is 120 cm^2^/gram, e.g., Pe = PS/S (Pardridge, 2016). Diazepam freely crosses the BBB, and the Pe value for diazepam approximates an upper limit of solute Pe at the BBB *in vivo*. The PS product for [^14^C]-diazepam *in vivo* in the rat is 21 uL/gram/sec (Tanaka and Mizojiri, 1999), which corresponds to a Pe value of 1.8 × 10^–4^ cm/sec, after correction for the brain capillary surface area *in vivo*. The PS value for [^14^C]-sucrose *in vivo* in the rat is 0.4 uL/min/gram (Miah et al., 2017a, b), which corresponds to a Pe value of 5.5 × 10^–8^ cm/sec. However, the use of radiolabeled sucrose to measure the Pe *in vivo* is problematic, owing to a small impurity of highly diffusible substances in commercially available sucrose preparations (Miah et al., 2017a, b). The PS value for [^13^C]-sucrose, a non-radioactive form of sucrose, can be measured by liquid chromatography-mass spectrometry (LC-MS), which eliminates the role of small amounts of impurities in the measurement of the BBB PS product for sucrose. The PS value for [^13^C]-sucrose, as measured by LC-MS, in the rat *in vivo* is 10-fold lower, 0.04 uL/min/gram, as compared to the PS value for [^14^C]-sucrose, as measured by radioactivity (Miah et al., 2017a, b). This sucrose PS value of 0.04 uL/min/gram corresponds to a Pe value for sucrose *in vivo* of 5.5 × 10^–9^ cm/sec. Therefore, the BBB Pe value for sucrose, 5.5 × 10^–9^ cm/sec, is 5 log orders of magnitude lower than the Pe value for diazepam, 1.8 × 10^–4^ cm/sec. The 10-fold over-estimation of the Pe value, using radiolabeled sucrose, would be expected if there was a 1% impurity which had a BBB Pe value 3 log orders of magnitude higher than the Pe of sucrose, which would still be 2 log orders of magnitude lower than the Pe value for diazepam. The role of the impurity in the estimation of the sucrose Pe value in an *in vitro* BBB model is less significant, owing to the higher Pe for sucrose with *in vitro* BBB models. If the Pe value for sucrose *in vitro* was 10-fold higher, e.g., 5.5 × 10^–8^ cm/sec, then the presence of the highly diffusible impurity would only result in a doubling of the Pe value measured *in vitro*. The use of the LC-MS and [^13^C]-sucrose methodology for *in vitro* BBB models would provide little value over the current use of radiolabeled sucrose, because, as discussed below, the sucrose Pe value, even in the most developed *in vitro* BBB model, is several log orders of magnitude higher than the Pe value for sucrose *in vivo*.

The sucrose Pe value in a Transwell co-culture is 5 × 10^–6^ cm/sec (Coisne et al., 2005). Similarly, the sucrose Pe value in a dynamic *in vitro* BBB model with astrocyte co-culture and a flow system is 5 × 10^–6^ cm/sec (Santaguida et al., 2006). The sucrose Pe value with these *in vitro* BBB models is 3 log orders of magnitude higher than the sucrose Pe value *in vivo* (Miah et al., 2017a, b). The sucrose Pe value with an *in vitro* BBB model derived from RA-activated hPSC cells is 5.3 × 10^–7^ cm/sec (Lippmann et al., 2014). In this latter model, the TEER values reach 3000 ohm⋅cm^2^ (Lippmann et al., 2014), yet the Pe value for sucrose is still 100-fold higher than the Pe value for sucrose *in vivo* (Miah et al., 2017a, b). These findings indicate that even the best *in vitro* BBB models with high TEER values do not replicate the permeability properties of the BBB *in vivo*. This is attributed to the significant down-regulation of BBB-related gene expression when brain endothelial cells are grown in cell culture.

### BBB-Specific Gene Expression With *in vitro* BBB Models

The best *in vitro* BBB models, based on high TEER values, are still 100-fold leaky to sucrose compared to the sucrose Pe *in vivo*. The difficulty over the last 40 years in the establishment of an *in vitro* BBB model that replicates the BBB *in vivo* may be a function of the profound down-regulation of BBB-specific gene expression that takes place when brain endothelial cells are removed from the complex architecture of the NVU (Figure 2A) and grown in cell culture. The measurement of gene expression with *in vitro* BBB models should be compared to gene expression in freshly isolated brain microvessels or microvascular endothelium. Gene expression was measured in freshly isolated rat brain microvessels and compared to gene expression in a primary culture of rat brain endothelial cells, and BBB-related gene expression was decreased by 1 to 3 log orders of magnitude in the primary culture of brain endothelial cells (Calabria and Shusta, 2008). Gene expression was measured in either a primary culture of human brain endothelial cells, or in the hCMEC/D3 line of human brain endothelial cells (Urich et al., 2012). Endothelial cell RNA was hybridized to Affymetrix GeneChip 3’ IVT Express system, and gene expression in the cultured endothelium was compared to the previously reported transcriptome of FACS purified mouse endothelial cells (Daneman et al., 2010). The expression of many BBB transporter members of the SLC or ABC gene families was down-regulated in culture by up to 6 log orders of magnitude compared to brain capillary endothelial gene expression *in vivo* (Urich et al., 2012).

### BBB on a Chip *in vitro* Models

A novel variation of the cell culture or *in vitro* BBB model is the “BBB on a chip,” where a silicone chip is fabricated to allow for medium flow through an outer endothelial compartment, with astrocytes cultured in an inner compartment, thus establishing a “microfluidic” form of the *in vitro* BBB model. Endothelial cells and astrocytes were originally co-cultured on a silicon ship by Ma et al. (2005). This model had no flow component and it was found that if the size of the channel connecting the endothelial and astrocyte compartments was 0.4 micron, there was no spread of astrocyte foot processes into the endothelial chamber. This result confirmed the earlier work of Hurwitz et al. (1993). Advances in the engineering of silicone chips allowed for fluid flow through the endothelial chamber (Booth and Kim, 2012), which creates a shear stress upon the endothelial wall that attempts to mimic the endothelial shear stress *in vivo*. In this respect, the microfluidic BBB on a chip model is an extension of the Dynamic *In Vitro* (DIV) model of Santaguida et al. (2006). The hypothesis underlying the development of dynamic or microfluidic *in vitro* BBB models is that BBB properties of the brain endothelium are induced by shear stress of capillary blood flow. One argument against this hypothesis is that endothelia in capillaries of peripheral organs are also subjected to flow-induced shear stress, and these endothelia do not exhibit the barrier properties of the cerebral capillary endothelium. The microfluidic silicone chips are now commercially available. In one model, hCMEC/D3 endothelial cells are cultured in the outer flow chamber and rat brain astrocytes are cultured in the inner chamber and the flow rate of 5 uL/min produces a shear stress of about 3 dyne/cm^2^ (Brown et al., 2019). This shear stress approximates the shear stress in brain capillaries *in vivo*, which was measured with a brain cranial window (Mairey et al., 2006). Brain capillary flow rates were measured by tracking red blood cell velocity, and the capillary brain blood flow rate was about 500 microns/sec, which produced a shear stress of 5-20 dyne/cm^2^ (Mairey et al., 2006). The presence of the medium flow increases resistance across the endothelial layer in the DIV model (Santaguida et al., 2006), and increases tight junctional protein expression in the endothelium in the microfluidic model (Brown et al., 2019). However, dynamic or microfluidic *in vitro* BBB models are still leaky compared to the BBB *in vivo*. The sucrose Pe in the dynamic *in vitro* BBB model (Santaguida et al., 2006) is 3 log orders higher than the sucrose Pe at the BBB *in vivo* (Miah et al., 2017a, b). Similarly, the microfluidic *in vitro* BBB on a chip model is leaky compared to the BBB *in vivo*. The Pe value for 10 kDa dextran is 1.5 × 10^–5^ cm/sec (Brown et al., 2019), which is 4 log orders of magnitude higher than the Pe value for sucrose *in vivo*, 5.5 × 10^–9^ cm/se (Miah et al., 2017a, b). These considerations suggest the presence of a flowing medium in either the DIV or the microfluidic *in vitro* BBB model produce an *in vitro* BBB model that is leaky compared to the BBB *in vivo*. Apart from the presence of flow, the BBB on a chip model has the potential for direct astrocyte contact with the endothelium, since the outer and inner chambers are connected by 3 micron channels (Prabhakarpandian et al., 2013; Deosarkar et al., 2015), and astrocyte foot processes can extend through 3 micron pores, but not 0.4 micron pores (Hurwitz et al., 1993). However, the channels in the chip models are relatively long, 100 microns (Deosarkar et al., 2015), as compared to the much thinner thickness, 10 microns, of typical Trans-well chambers. Fluorescent microscopy shows the extension of astrocyte foot processes through the 100 micron channels of the chip falls off significantly over the length of the 100 micron channel (Deosarkar et al., 2015).

In summary, even current *in vitro* BBB models, which are much improved over earlier *in vitro* models, still do not replicate the BBB *in vivo*, with respect to permeability properties, as exemplified by the sucrose Pe value, or with respect to gene expression, as shown by comparison of the BBB transcriptome in cell culture as compared to freshly isolated brain endothelial cells. It is still not clear if the induction of BBB gene expression in endothelial cells by astrocytes, or other cellular components of the NVU, is mediated by soluble diffusible factors or by cell to cell contact with the capillary endothelium.

## Experimental Limitations of Isolated Brain Microvessel Preparation

There are limitations of the isolated brain microvessel model, but these limitations can be addressed experimentally. Metabolic studies in the freshly isolated brain microvessel are limited owing to ATP depletion (Lasbennes and Gayet, 1984). However, brain microvessels still take up amino acids with a pattern that resembles BBB transport *in vivo* (Table 2). Microvessels are comprised of both luminal and abluminal endothelial membranes, and both membranes are exposed in the isolated brain capillary preparation (Hjelle et al., 1978). Amino acid transport across the luminal and abluminal membranes can be dissected by first isolation of brain capillary plasma membranes followed by density separation of luminal vs abluminal membranes (Sanchez del Pino et al., 1992). Brain microvessels are comprised of multiple cell types, including endothelium, pericytes, smooth muscle cells, astrocyte endfeet, and neuronal processes. The relative abundance of these cells in the microvessel preparation has been estimated with QTAP measurements of cell specific proteins (Table 1). Individual cells comprising the brain microvessel can be separated by FACS methodology (Agarwal and Shusta, 2009; Daneman et al., 2010; Chasseigneaux et al., 2018; Munji et al., 2019). Brain microvessels are typically isolated from brain homogenate by a dextran density centrifugation method, and this approach has been incorporated into *in vivo* measurements of BBB transport of biologics using the capillary depletion method (Triguero et al., 1990). Following the intra-arterial or intravenous injection of radiolabeled large molecule drugs, the transport of the drug through the BBB and into brain parenchyma is confirmed with the capillary depletion method, which separates the vascular pellet from the post-vascular supernatant. If the drug has a volume of distribution (VD) in the post-vascular supernatant higher than a blood volume marker such as albumin, then it can be inferred the drug has crossed the BBB (Triguero et al., 1990). Conversely, if a drug, e.g., acetylated low density lipoprotein, which is a ligand for the BBB scavenger receptor, is only endocytosed into the endothelium, without exocytosis into brain interstitial fluid, then the VD in the post-vascular supernatant is only equal to the VD of the blood volume marker (Triguero et al., 1990), and this indicates the ligand is trapped in the vascular endothelial compartment without transcytosis through the BBB. The capillary depletion method should be used only to investigate BBB transport of ligands that are bound to the BBB receptor with high affinity, e.g., low nM dissociation constant (KD) of binding. It is this high affinity binding to the endothelial receptor that causes retention of the ligand in the vascular pellet during the 4°C homogenization and centrifugation procedure (Triguero et al., 1990). Conversely, if the ligand binds to the BBB receptor with low affinity, e.g., with a KD of uM, then rapid dissociation of the endothelial bound ligand will take place during the capillary depletion process. This rapid dissociation will produce an artifactual elevation of the VD in the post-capillary supernatant, and lead to the erroneous conclusion that the ligand crosses the BBB *in vivo*.

## Conclusion and *in vitro*−*in vivo* Correlations

The isolated brain microvessel is a robust experimental model of either the BBB or the NVU which can be applied in many experimental applications, including proteomics, genomics, *ex vivo* transport and receptor binding, and the development of *in vitro* models of BBB transport. An important, yet under-developed application of the isolated brain microvessel is the extension of this model to human neurological disease. Microvessels can be isolated from fresh or frozen autopsy human brain. This would allow for the investigation of the role certain BBB transporters play in the development of human neurologic disease. For example, mutations in the LAT1 (SLC7A5) gene play a role in the development of autism spectrum disorders (Tarlungeanu et al., 2016). Brain microvessels have been removed from fresh human brain removed at neurosurgery for cerebral dysplasia (Shusta et al., 2002a). Although there are few clinical applications where significant amounts of fresh human brain can be removed at neurosurgery, microvessels can be isolated from fresh human brain tumors, which are removed in bulk at neurosurgery.

The extrapolation of experimental findings produced with isolated brain microvessels to the *in vivo* condition requires careful *in vitro*/*in vivo* correlation studies. For example, following the identification of certain SLC or ABC transporters with QTAP studies of brain microvessels, additional investigations are necessary to localize the SLC or ABC transporter to the endothelium, as opposed to the other cells that make up isolated brain capillaries, e.g., pericytes, smooth muscle cells, astrocyte foot processes, and nerve endings. Following the localization of a given SLC or ABC transporter at the endothelium, it is necessary to further localize the transporter expression at the luminal or abluminal endothelial membrane.

The localization of a given SLC or ABC transporter to the brain microvessel should be correlated with nutrient or drug transport across the BBB *in vivo*. For example, ENT purine nucleoside transporters may be localized to the brain microvessel. However, as discussed above, BBB transport of nucleoside transport *in vivo* is characterized by: (a) selective purine nucleoside transport with minimal pyrimidine transport, and (b) sodium dependency. Only these properties are exhibited by the CNT2-type purine nucleoside transporter.

The need for *in vitro*/*in vivo* correlation is most crucial in the investigation of “*in vitro* BBB” models. As discussed above, there is a profound down-regulation of BBB-specific gene expression in cultured endothelium as compared to gene expression in brain endothelial cells *in vivo*. This down-regulation of BBB gene expression in cell culture manifests by reduction of CMT-mediated solute transport *in vitro*, as compared to solute transport *in vivo*, and a leakiness to poorly diffusible solutes such as sucrose in the *in vitro* model, as compared to sucrose transport across the BBB *in vivo*. *In vivo*/*in vitro* comparisons are important in the analysis of *in vitro* BBB models, and the sole reliance on drug transport across the *in vitro* BBB model may hinder future development of BBB-penetrating pharmaceuticals.

## Author Contributions

The author confirms being the sole contributor of this work and has approved it for publication.

## Conflict of Interest

WP was consultant to the ArmaGen, Inc.
