# Supplementary material for: The Isolated Brain Microvessel: A Versatile Experimental Model of the Blood-Brain Barrier
Source: Front Physiol. 2020 May 7;11:398. doi: 10.3389/fphys.2020.00398 (PMC7221163; doi:10.3389/fphys.2020.00398)
Supplement: Supplementary file 1 [file Table_1.docx]

**Supplementary Table 1.** Transcriptome derived from suppression subtractive hybridization of cDNA libraries produced from freshly isolated rat or human brain microvessels

| **No.** | **SSH ID^1^** | **Gene** | **Name** | **Original accession no ^1^** | **Updated accession no.** |
| --- | --- | --- | --- | --- | --- |
| *Transporters* | | | | | |
| 1 | LK42 | Slco1a4 | Rat organic anion transporting polypeptide 2 (oatp2) | U88036 | NM_131906 |
| 2 | LK34 | Tfrc | Rat transferrin receptor 1 | M58040 | NM_022712 |
| 3 | LKH17 | Slco1c1 | Rat BBB specific anion transporter 1 (Bsat1/Oatp14) | NM_053441 | NM_053441 |
| 4 | LKM98 | Slc7a1 | Rat cationic amino acid transporter 1 (Cat1) | NM_013111 | NM_013111 |
| 5 | hLK61 | SLC16A1 | Human monocarboxylic acid transporter 1 (MCT1) | AL162079 | NM_003051 |
| 6 | LKM71 | Slc39a10 | Rat zinc transporter ZIP10 | BM382815 (EST) | XM_006244938 |
| 7 | LKH20 | Cav1 | Rat caveolin-1α | AB029929 | NM_031556 |
| 8 | LKM87 | Fxyd5 | Rat FXYD domain-containing ion transport regulator 5 | NM_021909 | NM_021909 |
| 9 | LKH60 | Atp1a2 | Rat ATPase Na+/K+ transporting subunit alpha 2 | NM_012505 | NM_012505 |
| 10 | LK31 | Atp1b2 | Rat ATPase Na+/K+ transporting subunit beta 2 | AA817814^2^ (EST) | XM_006246581 |
| *Vascular Remodeling* | | | | | |
| 11 | LK7 | Flt1/VEGFR | Rat FMS-related tyrosine kinase 1 | D28498 | NM_019306 |
| 12 | LKM31 | Ptprb/  VE-PTP | Rat receptor-type tyrosine-protein phosphatase beta | AF157628 | NM_001372134 |
| 13 | hLK20 | SPARC | Human secreted protein acidic cysteine rich | XM_003989 | NM_003118 |
| 14 | LKM16 | Adgrf5 | Rat adhesion G protein-coupled receptor F5 | BM382810 (Novel) | XM_006244597 |
| 15 | LKM86 | Fgd5 | Rat FYVE, RhoGEF and PH domain containing 5 | BM382816^2^ (Novel) | NM_001108637 |
| 16 | LKM36 | Apold1 | Rat apolipoprotein L domain containing 1 | BM382813^2^ (Novel) | NM_001003403 |
| *Junctional Proteins* | | | | | |
| 17 | LKM88 | Esam | Rat endothelial cell adhesion molecule | AF361882 | NM_001004245 |
| 18 | LKM82 | Prom1 | Rat prominin 1 | AF386758 | NM_021751 |
| 19 | LKM20 | Pecam1 | Rat platelet and endothelial cell adhesion molecule 1 | U77697 | NM_031591 |
| 20 | hLK5 | CLDN5 | Claudin-5 | XM_009839 | NM_003277 |
| 21 | LKM34 | Jcad | Rat junctional cadherin 5 associated | BM382812 (Novel) | XM_001056659 |
| 22 | LK29 | Gjc1 | Rat gap junction protein, gamma 1 | X63100 | XM_017597050 |
| *Amyloid-Related Genes* | | | | | |
| 23 | LKM1 | Aplp2 | Rat amyloid beta precursor like protein 2 | X77934 | NM_012906 |
| 24 | LKH24 | Itm2a | Rat integral membrane protein 2A | NM_008409 | NM_001025712 |
| 25 | LKM43 | Serpine2 | Rat serpin family member 2 | NM_009255 | NM_019197 |
| *Growth Factors* | | | | | |
| 26 | LK6 | Igf2 | Rat insulin-like growth factor 2 | X14834 | NM_031511 |
| 27 | hLK28 | IGFBP3 | Human insulin-like growth factor binding protein 3 | M31159 | NM_000598 |
| 28 | hLK23 | TMEFF2 | Human transmembrane protein with EGF like and two follistatin like domains 2 (tomoregulin) | AB004064 | NM_016192 |
| 29 | hLK4 | PTN | Human pleiotrophin (human nerve growth factor-1) | M57399 | BC005916 |
| 30 | hLK40 | FGF19 | Human fibroblast growth factor 19 | AF110400 | NM_005117 |
| 31 | hLK24 | ARRDC2 | Human arrestin domain containing 2 | AF131826 | NM_001286826 |
| *Signal Transduction* | | | | | |
| 32 | LKM59 | Dusp1 | Rat dual specificity phosphatase 1 | U02553 | NM_053769 |
| 33 | LKM84 | Ptgds | Rat prostaglandin D2 synthase | NM_013015 | NM_013015 |
| 34 | LK25 | S100b | Rat S100 calcium binding protein B | S53527 | NM_013191 |
| 35 | hLK26A | GAB2 | Human GRB2 associated binding protein 2 | AB018413 | NM_080491 |
| 36 | LK1 | Apcdd1 | Rat APC down-regulated 1 | AI072079  (EST) | XM_001071384 |
| 37 | LKM68 | Prkar1a | Rat protein kinase cAMP-dependent type 1 regulatory subunit alpha | BG671327  (EST) | XM_017597047 |
| 38 | hLK39 | NET1 | Human neuroepithelial cell transforming 1 | AW263232  (EST) | NM_005863 |
| *Hemostasis* | | | | | |
| 39 | LKH31 | VWF | Rat von Willebrand factor  (human homologue) | NM_000552 | NM_000552 |
| 40 | LKM19 | tPA | Rat tissue plasminogen activator | NM_013151 | NM_013151 |
| *Secretion* | | | | | |
| 41 | LKH37 | Cpe | Rat carboxypeptidase E | NM_013128 | NM_013128 |
| 42 | LKM6 | Srgn | Serglycin | NM_020074 | NM_020074 |
| *Pseudogenes* | | | | | |
| 43 | LK36 |  | Rat MHC class I pseudogene | L40364 | NR_002597 |
| *Transcription Factors* | | | | | |
| 44 | LKM96 | Eloa | Rat elongin A | NM_017103 | NM_017103 |
| 45 | LKM44 | Erg | Rat ETS transcription factor ERG | AB031088 | NM_133397 |
| 46 | LK41 | Ezh1 | Rat enhancer of zeste 1 polycomb repressive complex 2 subunit  (Mouse homologue) | NM_007970 | NM_007970 |
| 47 | LK27 | Nfkbia | Rat NFKB inhibitor alpha | X63594 | NM_001105720 |
| 48 | LK37 | Smarca2 | Rat SWI/SNF related, matrix associated, actin dependent regulator of chromatin, subfamily a, member 2 | X72889 | XM_032891750 |
| 49 | LK16 | Btg2/PC3 | Rat BTG anti-proliferation factor 2 | M60921 | NM_017259 |
| 50 | LKM71 | Slfn5 | Rat schlafen family member 5 | BM382815 (Novel) | XM_001081036 |
| 51 | LKM92 | Epas1 | Rat endothelial PAS domain protein 1 | NM_023090 | NM_023090 |
| 52 | hLK22 | HNRNPDL | Human heterogenous nuclear ribonucleoprotein D like | D89678 | NM_031372 |
| *Unknown Function* | | | | | |
| 53 | LKH15 | Cyyr1 | Rat cysteine and tyrosine rich 1 | BM382807^2^ (Novel) | NM_001013980 |
| *Myelin Related Proteins* | | | | | |
| 54 | LKH5 | Mbp | Rat myelin basic protein | NM_017026 | NM_017026 |
| 55 | LKM7 | Mpzl1 | Rat myelin protein zero like 1  (Human homologue) | NM_003953 | NM_003953 |
| 56 | LKM4 | Plp1 | Rat proteolipid protein 1 | NM_030990 | NM_030990 |
| *Pericyte Related Proteins* | | | | | |
| 57 | LKM17 | Pdgfrb | Rat platelet derived growth factor receptor beta | NM_008809 | NM_031525 |
| 58 | LKH11 | Rgs5 | Rat G protein signaling regulator 5 | U67188 | XM_032915287 |
| 59 | LKM8 | Gpcpd1 | Rat glycerophosphocholine phosphodiesterase 1 | BM382808^2^ (Novel) | NM_198779 |
| *Astrocyte Related Proteins* | | | | | |
| 60 | LKM65 | Gfap | Rat glial fibrillary acidic protein | AF028784 | NM_017009 |
| *Cytoskeleton* | | | | | |
| 61 | LKM91 | Myl9/Mlc20 | Rat myosin light chain 9 | S77900 | NM_001100885 |
| 62 | LKH42 | Vim | Rat vimentin | NM_031140 | NM_031140 |
| 63 | LK24 | Utrn | Rat utrophin | AJ002967 | NM_013070 |
| 64 | LKM49 | Flna | Rat filamin A | BI285511 (EST) | NM_001134599 |
| 65 | LKM63 | Fhl3 | Rat four and a half LIM domains 3 | AI171222 (EST) | XM_008764017 |
| 66 | LK19 | Vcl | Rat vinculin | L18880 | NM_001107248 |
| *Lipid Related Proteins* | | | | | |
| 67 | LKH8 | Pltp | Rat phospholipid transfer protein | NM_011125 | NM_001168543 |
| 68 | LKM39 | Scd2 | Rat stearoyl-coenzyme A desaturase 2 | NM_031841 | NM_031841 |
| 69 | hLK9 | PON2 | Human paraoxonase 2 | NM_000305 | NM_000305 |
| 70 | hLK19 | A2M | Human alpha-2 macroglobulin | NM_000014 | NM_000014 |
| *Basement Membrane Proteins* | | | | | |
| 71 | hLK32 | NID1 | Human nidogen 1 | AV706417  (EST) | NM_002508 |

^1^From Li et al (2001) and Li et al (2002) for rat brain capillary genomics and from Shusta et al (2002) for human brain capillary genomics. EST=expressed sequence tag.

^2^Reverse complement sequence
